# Genome Sequence Variations of Infectious Bronchitis Virus Serotypes From Commercial Chickens in Mexico

**DOI:** 10.3389/fvets.2022.931272

**Published:** 2022-07-12

**Authors:** Henry M. Kariithi, Jeremy D. Volkening, Christina M. Leyson, Claudio L. Afonso, Nancy Christy, Eduardo Lucio Decanini, Stéphane Lemiere, David L. Suarez

**Affiliations:** ^1^Exotic and Emerging Avian Viral Diseases Research Unit, Southeast Poultry Research Laboratory, U.S. National Poultry Research Center, USDA-ARS, Athens, GA, United States; ^2^Biotechnology Research Institute, Kenya Agricultural and Livestock Research Organization, Nairobi, Kenya; ^3^BASE_2_BIO, Oshkosh, WI, United States; ^4^Boehringer Ingelheim Animal Health, Guadalajara, Mexico; ^5^Boehringer Ingelheim Animal Health, Lyon, France

**Keywords:** lineage, NGS, mutation, recombination, hypervariable region, vaccine

## Abstract

New variants of infectious bronchitis viruses (IBVs; *Coronaviridae*) continuously emerge despite routine vaccinations. Here, we report genome sequence variations of IBVs identified by random non-targeted next generation sequencing (NGS) of vaccine and field samples collected on FTA cards from commercial flocks in Mexico in 2019–2021. Paired-ended sequencing libraries prepared from rRNA-depleted RNAs were sequenced using Illumina MiSeq. IBV RNA was detected in 60.07% (*n* = 167) of the analyzed samples, from which 33 complete genome sequences were *de novo* assembled. The genomes are organized as 5'UTR-[Rep1a-Rep1b-S-3a-3b-E-M-4b-4c-5a-5b-N-6b]-3'UTR, except in eight sequences lacking non-structural protein genes (accessory genes) 4b, 4c, and 6b. Seventeen sequences have auxiliary S2' cleavage site located 153 residues downstream the canonically conserved primary furin-specific S1/S2 cleavage site. The sequences distinctly cluster into lineages GI-1 (Mass-type; *n* = 8), GI-3 (Holte/Iowa-97; *n* = 2), GI-9 (Arkansas-like; *n* = 8), GI-13 (793B; *n* = 14), and GI-17 (California variant; CAV; *n* = 1), with regional distribution in Mexico; this is the first report of the presence of 793B- and CAV-like strains in the country. Various point mutations, substitutions, insertions and deletions are present in the S1 hypervariable regions (HVRs I-III) across all 5 lineages, including in residues 38, 43, 56, 63, 66, and 69 that are critical in viral attachment to respiratory tract tissues. Nine intra-/inter-lineage recombination events are present in the S proteins of three Mass-type sequences, two each of Holte/Iowa-97 and Ark-like sequence, and one each of 793B-like and CAV-like sequences. This study demonstrates the feasibility of FTA cards as an attractive, adoptable low-cost sampling option for untargeted discovery of avian viral agents in field-collected clinical samples. Collectively, our data points to co-circulation of multiple distinct IBVs in Mexican commercial flocks, underscoring the need for active surveillance and a review of IBV vaccines currently used in Mexico and the larger Latin America region.

## Introduction

Infectious bronchitis virus (IBV; type species of the *Coronaviridae* family) causes the acute, highly contagious avian infectious bronchitis (IB) disease that primarily affects the upper respiratory tract in chickens of any age, but which can also cause urogenital or enteric disease, resulting in decreased production depending on virus strain and co-infecting pathogens, age, vaccination history, and immune competency of chickens (1, 2). The virus is shed by naturally infected and/or vaccinated birds and is transmitted via respiratory discharges (acute phase) and feces (disease recovery phase) to susceptible naïve birds (3). After initial respiratory tract infection, the virus can be disseminated to other tissues including trachea, lungs, kidney, oviduct, alimentary, and proventriculus (2).

The non-segmented, positive single-stranded RNA genome (~ 27.6 kb in size) of IBV, which can also serve as a viral mRNA, comprises six genes flanked by 5′-/3′- untranslated regions (UTRs) (4, 5). Occupying about two thirds of the genomic 5′-end, gene 1 encodes the replicase/polymerase complex 1a and 1ab (Rep1a/1ab), which is produced by a programmed−1 ribosomal frameshifting mechanism that allows continuation of translation beyond the Rep1a stop codon. Rep1a/ab is proteolytically cleaved into 15 non-structural proteins by the virally-encoded accessory gene 3 (papain-like protease; PL ^pro^) and gene 5 (3-C-like protease; 3CL ^pro^) proteases (6, 7). Gene 2 encodes spike (S) glycoprotein, the largest structural and most divergent of all IBV proteins, which is proteolytically cleaved by cellular furin protease into subunits S1 and S2 (8). Genes 3 and 4 encode two accessory proteins and one membrane-binding structural protein each [3a/3b, and envelope (E), and 4b/4c, and membrane (M), respectively]. Gene 5 encodes accessory proteins 5a and 5b, while gene 6 encodes the structural nucleocapsid (*N*) protein and accessory protein 6. Accessory genes 3a, 3b, 4b, 4c, 5a, 5b, and 6b, which have a wide range of genomic configurations, are non-essential for virus replication (9). Accessory genes 4b, 4c, and 6b are rarely reported in the literature despite being present in many unpublished IBV genome sequences, but little is known about their roles in viral replication or pathogenesis (10, 11).

Whereas, spike subunit S2 is highly conserved amongst IBVs, the hypervariable regions (HVRs I-III) of the S1 harbor the majority of nucleotide (nt) heterogeneity between different strains (12, 13). Subunit S1 also contains the receptor-binding domain (RBD), which is essential for entering susceptible chicken cells and induction of host immune responses (14). New IBV variants, which can evade vaccine-induced immunity, continually emerge due to the mutations (caused by replication errors), and/or recombination events (caused by template switching) in the S1 sequences (15–17). Due to its close correlation with IBV serotypes, S1 sequence is used to classify IBV into 7 genogroups (GI-GVII) comprising at least 32 distinct lineages and several unassigned inter-lineage recombinants (1, 9, 18–20). “Genogroup” represents stable IBV categories and “lineage” is a descriptive dynamic serotype grouping (21).

The Mass-type serotype was first identified in the USA in the 1930s (22), but IBVs are now globally distributed in poultry, with regional-specific genotypes and serotypes that are often closely related to the live attenuated vaccine strains used in the regions, or are unique variants (15, 18, 23–25). Serotypes belonging to lineage GI-11 [South American 1 (SAI) serotypes] and lineage GI-16 (Q1-like serotypes) extensively circulate in South American chicken flocks (21). The uniquely South American SAI serotypes, which are associated with respiratory/enteric disease and reduced egg production, emerged in the 1950s in commercial poultry flocks in Brazil, and later spread to Argentina and Uruguay (18, 25). The Q1-like serotypes, which emerged in late 1970s in Asia, are more widespread than the SAI serotypes (18, 21). Both the SAI and Q1-like serotypes have low antigenic relatedness with the Mass-type vaccine strains (lineage GI-1) that are extensively used vaccination programs globally (16, 21). Other serotypes reported in South America include lineage GI-13 (793B or 4/91-like) in Brazil, Chile and Honduras (26–28). In Mexico, Mass-type (lineage GI-1), Holte/Iowa-97 (BL-56; lineage GI-3), Ark (lineage GI-9), and Q1-like serotypes have been reported in commercial flocks (29–31).

Live-attenuated (derived from virulent strains attenuated via serial passage in chicken eggs) or inactivated vaccines are routinely used in IB control (32–34). Routine use of serotype-specific live vaccines can result in evolution of novel variants (due to mutations, insertions, deletions, or recombination between co-infecting field and vaccine strains) that are serologically distinct from the vaccine strains, with potential negative impacts on vaccine efficacy (35–37). The apparent regionality of IBV diversity underscores the importance of viral characterization, which can be used to assess and properly deploy existing vaccines and potentially identify when new vaccines need to be developed.

In the current study, we sequenced 30 complete IBV genome sequences from clinical field samples collected from commercial flocks in Northern, Central and Southern Mexico in 2019–2021, along with 3 vaccine samples. We performed comparative analysis of sequence variation (vaccine vs. field sequences) and phylogenetic relationships with other IBVs, and assessed potential recombination events, point mutations, insertions, and deletions in the HVRs of the S glycoprotein. The data presented here expand current knowledge of the IBVs circulating in Mexico, which can inform vaccination strategies to control IB outbreaks in the country and in Latin America.

## Materials and Methods

### Type, Origin, and Processing of Samples

The samples used in this study were randomly collected from “apparently healthy” (i.e., no observable clinical signs of disease at the time of sampling) commercial broiler (aged 21–42 days) and layer (aged 7 weeks) chicken flocks in central, northern, and southern regions of Mexico between April 2019 and December 2021. The samples were derived from respiratory (choana and lung), immunological (spleen and bursa) and digestive (cloaca) tissues from 100 birds per flock using sterile flocked swabs and pooled (25 samples per pool) in sterile 1.5 mL viral transport media. Samples were then spotted on 4-sample-area (125 μl per area) Whatman Flinders Technology Associates (FTA) cards® (Millipore-Sigma) within 24 h of collection and dried for at least 2 h at room temperature (RT; 15–25°C). After drying, each sample-spotted FTA card was individually enclosed in double leak-proof zip-lock plastic bags with Whatman desiccant packets (GE healthcare). In addition to field samples, three vaccine samples (i.e., live attenuated Mass-type, 4/91 variant and Mass-type/Connecticut recombinant strains) from Boehringer Ingelheim Animal Health (BIAH), Mexico, were also FTA-spotted. The detailed information about what flocks were vaccinated and with what type of vaccine is not publicly available because of propriety and confidentiality between BIAH and their clients. All samples were shipped to the Southeast Poultry Research Laboratory (SEPRL), USDA-ARS, Athens, GA, and stored at −80°C in a BSL-3 laboratory until further processing.

### RNA Extraction

From each sample-spotted FTA card, 24, 3-mm disks (i.e., 6 disks per spotted area) were punched out using sterile disposable biopsy punches (Robbins Instruments, USA) and incubated for 30 min at room temperature in 240 μL of nuclease-free TE buffer (10 mM Tris-HCl; 0.1 mM EDTA, pH 8.0) to elute nucleic acids. Total RNA was extracted from 100 μL of the TE eluate using MagMAX™-96 AI/ND Viral RNA Isolation Kit (Thermo Fisher Scientific, MA, USA) on an automated KingFisher Magnetic Particle Processor (Thermo Fisher, USA) following manufacturer's instructions. To selectively deplete abundant host-specific RNAs (18S, 28S and mitochondrial) and bacterial rRNAs (16S/23S), extracted RNAs were treated with an in-house RNaseH host rRNA depletion protocol we have recently described (38).

### Library Preparation and NGS

Sequencing libraries were prepared using sequence-independent, single-primer amplification (SISPA) as previously described (39). Briefly, cDNAs were synthesized from 10 μL of RNA using random K-8N primer with SuperScript ^TM^ IV First Strand synthesis Kit (Invitrogen, USA) and Klenow polymerase (NEB Inc., USA) kits, and then purified using Agencourt AMPure XP beads (Beckman Coulter Life Sciences, USA). Purified cDNAs were amplified by Phusion® High-Fidelity PCR Kit (NEB Inc., USA), and used to prepare sequencing libraries by the Nextera ^TM^ DNA Flex kit (Illumina, USA), which were then quantified by Qubit™ dsDNA HS Assay Kit (Thermo-Fisher Scientific) and Agilent 4150 TapeStation HS D5000 System (Agilent Technologies, Inc.). Based on their concentrations and average fragment sizes, equimolar concentrations (4 nM, 8 μL of each library) of the libraries were pooled, then digested by incubation with 0.2 N NaOH (5 min at RT). Pooled libraries were further diluted to 10 pM final concentration, a control library added (5% PhiX library v 3) and paired-end (2 × 300 bp) sequencing performed using a 600-cycle MiSeq Reagent Kit v3 (Illumina, USA). Each NGS run consisted of 48 multiplexed samples.

### Sequence Assembly

Customized workflows executed in Galaxy and Geneious Prime® 2021.2.2 platforms were used to assess the quality of the raw NGS reads (by FastQC) and trimming of adaptors (by Cutadapt) as previously described (40–43). Briefly, the host (chicken) and PhiX control reads were filtered out by mapping the trimmed reads against chicken (*Gallus gallus*) and PhiX174 reference genomes using BWA-MEM v 0.7.15.1 (44) with standard parameters. Trimmed/filtered overlapping forward and reverse read-pair sets were merged with PEAR v 0.9.6.1 (45); an in-house wrapper tool was then used to identify and remove chimeric Nextera reads. After digital normalization of the reads via k-mer abundances using khmer package (46), *de novo* sequence assembly was performed using MIRA v3.4.0 (47). BWA-MEM/samtools was used to re-call consensus sequences from the NGS reads aligned to the *de novo*-generated contigs (minimum coverage depth to call a base set at 3X).

### Sequence Annotation and Phylogenetic Analysis

Open reading frames (ORFs; used here to refer to contiguous nt stretches from start to stop codons without interrupting in-frame stop codons) in the assembled consensus sequences were determined and annotated using ORF Finder (minimum ORF size set at 50 NT including start and stop codons) within the Geneious Prime® v 2022.1.1 (https://www.geneious.com). ORFs were confirmed by comparative analyses with annotated coronavirus (CoV) genes and genomes available at GenBank and/or PubMed. Classification of the assembled sequences was based on Valastro et al. (18). Putative protein domains were predicted using translated protein sequences with InterProScan v 2.0 (48) executed in Geneious Prime. For confirmation, putative annotations were aligned with similar annotations (coding regions and domains/motifs of translated amino acid sequences) of a Geneious Prime local database (containing reference sequences retrieved from GenBank and/or PubMed using BLASTp algorithm). ProPserver v1.0 (49) was used to predict putative cleavage site of the S glycoprotein at the border between subunits S1 and S2 (R-X-X-R↓S motif), and in S2′ site (R-X-R↓S motif) located upstream of subunit S2 ectodomain. Asparagine (N)-linked glycosylation sites in S1 protein sequences were predicted using NetNGlyc-1.0 (www.cbs.dtu.dk/services/NetNGlyc/) only sites with scores of at least 0.6 and supported by six of the nine predictive neural networks of the server were accepted.

The genome and specific gene sequences obtained from this study, together with sequences of representative serotypes within the IBV lineages [retrieved from the GenBank; lineages based on current IBV classification system (18)] were used for multiple sequence alignment using MAFFT v 7.490 (50). To minimize effects of poorly aligned regions, the multiply aligned sequences were trimmed using trimAl tool v 1.3 (51) with gappyout mode. Phylogenetic analysis was performed using maximum likelihood method executed in MEGA with 1,000 bootstrap replicates of the original data and the best model automatically identified by the software (52).

### Analyses of Point Mutation, Insertions/Deletions, and Putative Recombination Events

Multiple alignments of amino acid sequences of subunit S1 HVRs I-III from this study, together with sequences of representative serotypes within IBV lineages, were analyzed for presence of deletions/insertions (hereafter abbreviated as indels) and point mutations. For recombination events analysis, SplitsTree5 v 5.0.0_alpha (53) was used to determine the likelihood of recombination events in the complete S-gene sequences. Recombination events were further examined using seven heuristic recombination detection algorithms (RDP, GENECONV, BootScan, MaxChi, Chimaera, SiSscan, and 3Seq) executed in the Recombination Detection Program 4 (RDP4) v4.101 software suite (54), at highest *p*-value of 0.05 with Bonferroni multiple correction and SEQ-GEN parametric data simulations. Confirmation of putative recombinant event was accepted only when the recombination breakpoints were detected by at least five of the seven algorithms, and with breakpoints of the transferred fragments (recombinant regions) supported by corrected *p*-values of ≤1 × 10–6.

## Results

### Sequencing Libraries and Sequencing Data

In this study, we analyzed three vaccine and 275 field samples derived from immunological (*n* = 126), respiratory (*n* = 141), digestive (*n* = 8) tissues, and the vaccine samples (*n* = 3). Average fragment length distribution of the adaptor-ligated libraries (TapeStation estimates) ranged from 425 to 608 bp, but actual post-NGS average fragment length distribution post-FastQC (excluding adaptor sequences) were shorter, a discrepancy attributable to the fact that shorter fragments tend to cluster more efficiently than longer fragments (55). Total trimmed/filtered read counts ranged from 16,541 to 1.3 million reads. Proportions of chicken-specific reads ranged from 0.82 to 77.8%, with only six samples having over 50% of the reads mapping to the host genome.

### Detection of Viral and Pathogenic Bacterial RNAs

IBV RNA was detected in 60.07% (*n* = 167) of the analyzed samples. Fifty-five of the samples (20 immunological tissue samples, 32 respiratory tissue samples, and the 3 vaccine samples) had enough IBV-specific reads to allow for assembly of complete or nearly-complete genome or *S*-gene consensus sequences (Supplementary Table 1). In addition to IBVs, 10 spleen/bursa and nine choanal/lung tissues contained RNA of avian viruses belonging to families *Astroviridae* (chicken astrovirus, serogroup 1b), *Birnaviridae* (infectious bursal disease virus, genogroup 2b), *Paramyxoviridae* (avian paramyxovirus type 1, subgenotype V.1), *Pneumoviridae* (avian metapneumovirus subtype A), *Reoviridae* (avian rotavirus serogroups A, D, and F), and *Picornaviridae* (avian nephritis virus, chicken gallivirus A, chicken megrivirus group C-3, and sicinivirus type A). Picornaviruses, and in particular SiV, were overrepresented (detected together with IBV in 73.68% of the 19 samples). In addition to viral agents, avian pathogenic bacterial species were detected in 29 out of the 55 samples, including *Bordetella, Enterococcus* spp, *Gallibacterium anatis, Salmonella enterica*, and *Streptococcus* spp.

### Assembly of IBV Genome Sequences

Thirty-three complete genome sequences, six partial genome sequences, three complete *S*-gene sequences, and 13 partial *S*-gene sequences of IBVs were assembled (Table 1). Further analyses were restricted to the 33 complete genome sequences. One sequence was obtained from a spleen/bursa tissue sample swabbed from a 7-week old layer chicken, while 21 and nine sequences were from choanal/lung and spleen/bursa tissue samples, respectively, swabbed from broiler chickens aged between 21 and 42 days. All 33 genome sequences were supported by sufficient read depths (median read depths of 10–1,175 X) and genome coverage (99.98–100%). However, 19 of the genome sequences missed short stretches at the 5′- and/or the 3′- termini, which is not unusual for randomly primed viral genome sequencing (56).

**Table 1:** Sequence assembly coverage of 33 complete genome sequences assembled in this study.

**Sequence**	**Sampling date**	**Sample origin**	**Flock (age)**	**Tissue**	**IBV-specific reads**	**Forward read quality^a^**	**Reverse read quality^a^**	**No. of IBV contigs**	**Coverage depth^a^**	**Reads used for consensus^b^**	**Consensus seq length (bases)^c^**	**Missing nt at 5'-end^d^**	**Missing nt at 3'-end^d^**	**percent coverage^e^**
Live Mass-type vaccine strain/1616/19	23/Apr/19	BIAH Mexico	BIAH Mexico	Vaccine	8,839	2|2|2|2|38	2|2|2|25|36	4	0|15|**25**|39|407	5,135	27,469			100.%
4/91 vaccine variant/1619/19	23/Apr/19	BIAH Mexico	BIAH Mexico	Vaccine	2,490	2|2|21|25|36	2|2|2|2|38	28	0|6|**10**|16|112	2,053	27,656			100%
Mass-type-Conn recombinant vaccine strain/1623/19	23/Apr/19	BIAH Mexico	BIAH Mexico	Vaccine	11,636	2|2|2|25|35	2|2|2|2|38	2	0|21|**32**|51|572	6,899	27,474			100%
ck/MEX/2353/20	25/Nov/20	South Mexico	Broiler (42D)	Respiratory	6,072	2|33|37|38|38	2|35|37|38|38	9	0|22|**40**|71|390	6,032	27,410	184	138	99.98%
ck/MEX/2354/20	25/Nov/20	South Mexico	Broiler (42D)	Immunological	18,184	2|34|37|38|38	2|36|37|38|38	1	0|66|**131**|240|862	18,184	27,410			100%
ck/MEX/2359/20	25/Nov/20	South Mexico	Broiler (42D)	Respiratory	182,811	2|36|37|38|38	2|37|38|38|38	4	0|455|***1054***|2053|15505	174,143	27,634			100%
ck/MEX/2360/20	25/Nov/20	South Mexico	Broiler (42D)	Immunological	18,162	2|37|38|38|38	2|35|37|38|38	10	0|39|**116**|218|1929	17,859	27,167		83	99.99%
ck/MEX/2523/21	28/Jan/21	Central Mexico	Broiler (29D)	Respiratory	25,972	2|36|37|38|38	2|37|37|38|38	8	0|62|**148**|346|2345	25,154	27,449	21	3	99.99%
ck/MEX/2562/21	25-Feb-21	Central Mexico	Broiler (28D)	Respiratory	10,978	2|27|36|37|38	2|32|37|38|38	6	0|49|**71**|107|913	10,978	27,671			100%
ck/MEX/2563/21	25-Feb-21	Central Mexico	Broiler (28D)	Immunological	24,308	2|30|37|37|38	2|35|37|38|38	2	0|79|**152**|291|2404	24,308	27,805			100%
ck/MEX/2592/21	Apr/21	South Mexico	Broiler (21D)	Respiratory	20,491	2|21|28|37|38	2|26|34|37|38	2	0|32|**52**|100|409	17,806	27,700			100%
ck/MEX/2598/21	Apr/21	North Mexico	Broiler (27D)	Immunological	3,289	2|31|37|37|38	2|25|35|37|38	19	0|11|**24**|43|182	3,289	27,095		122	99.99%
ck/MEX/2602/21	Apr/21	South Mexico	Broiler (21D)	Immunological	5,422	2|28|36|37|38	2|23|33|37|38	10	0|23|**43**|68|293	5,422	27,623		11	100%
ck/MEX/2721/21	06/Jun/21	North Mexico	Broiler (21D)	Respiratory	14,548	2|33|37|37|37	2|27|36|37|37	7	0|63|**119**|210|523	14,465	27,280	54		99.99%
ck/MEX/2723/21	06/Jun/21	North Mexico	Broiler (28D)	Respiratory	65,561	2|33|36|37|37	2|36|37|37|37	3	0|246|**500**|953|3343	65,351	27,694	25		99.99%
ck/MEX/2725/21	06/Jun/21	North Mexico	Broiler (21D)	Respiratory	15,345	2|17|20|34|37	2|21|28|36|37	4	0|62|**101**|150|1016	14,551	27,594			100%
ck/MEX/2731/21	23/Jun/21	Central Mexico	Broiler (23D)	Respiratory	2,306	2|18|21|35|37	2|22|31|37|37	35	0|6|**11**|20|387	2,207	27,189	192		99.99%
ck/MEX/2742/21	11/Jul/21	South Mexico	Broiler (28D)	Immunological	24,335	2|18|31|36|37	2|24|35|37|37	2	0|75|**149**|279|3047	23,808	27,625	9		100%
ck/MEX/2743/21	11/Jul/21	South Mexico	Broiler (28D)	Respiratory	32,724	2|32|36|37|37	2|34|37|37|37	6	0|127|**261**|467|1855	32,606	27,509			100%
ck/MEX/2748/21	15/Jul/21	South Mexico	Broiler (28D)	Respiratory	6,983	2|18|25|36|37	2|23|33|37|37	8	0|33|**60**|96|325	6,891	27,619		12	100%
ck/MEX/2752/21	20/Jul/21	South Mexico	Broiler (21D)	Respiratory	37,386	2|34|36|37|37	2|36|37|37|37	7	0|87|**310**|539|1686	37,229	27,022	180	59	99.99%
ck/MEX/2753/21	20/Jul/21	South Mexico	Broiler (28D)	Immunological	8,260	2|18|26|36|37	2|22|33|37|37	1	0|42|**64**|98|323	8,178	27,450	47		99.99%
ck/MEX/2754/21	20/Jul/21	South Mexico	Broiler (21D)	Respiratory	4,346	2|18|27|36|37	2|23|34|37|37	6	0|20|**35**|60|210	4,256	27,411			100%
ck/MEX/2818/21	9/10/21	North Mexico	Broiler (28D)	Respiratory	7,109	2|33|36|37|37	2|34|37|37|37	4	0|29|**51**|82|316	7,076	27,617			100%
ck/MEX/2819/21	9/10/21	North Mexico	Broiler (28D)	Immunological	8,690	2|33|36|37|37	2|33|36|37|37	3	0|36|**66**|122|449	8,635	27,638		17	99.99%
ck/MEX/2826/21	9/13/21	South Mexico	Broiler (28D)	Respiratory	18,838	2|33|36|37|37	2|35|37|37|37	1	0|73|**117**|212|1491	18,800	27,567	30	4	99.99%
ck/MEX/2833/21	9/27/21	North Mexico	Broiler (21D)	Respiratory	32,389	2|31|34|37|37	2|34|36|37|37	2	0|105|**203**|347|4082	30,900	27,694		7	100%
ck/MEX/2860/21	10/12/21	South Mexico	Broiler (28D)	Respiratory	60,382	2|23|35|37|37	2|31|36|37|37	3	0|278|**470**|747|2866	59,588	27,622	9		100%
ck/MEX/2930/21	23-Nov-21	Central Mexico	Broiler (21D)	Respiratory	22,389	2|33|37|37|37	2|36|37|37|37	14	0|54|**137**|260|883	21,704	27,719			100%
ck/MEX/2944/21	7-Dec-21	North Mexico	Broiler (28D)	Respiratory	57,769	2|31|35|37|37	2|35|37|37|37	1	0|237|**400**|654|3974	56,592	27,585	48		99.99%
ck/MEX/2956/21	14-Dec-21	South Mexico	Broiler (28D)	Respiratory	196,791	2|34|36|37|37	2|36|37|37|37	2	0|629|***1175***|2272|29429	193,735	27,697			100%
ck/MEX/2960/21	14-Dec-21	Central Mexico	Layer (7.6W)	Immunological	5,153	2|18|25|36|37	2|23|34|37|37	12	0|16|**31**|52|402	4,805	27,524		41	99.99%
ck/MEX/2961/21	15-Dec-21	Central Mexico	Broiler (21D)	Respiratory	19,584	2|19|32|37|37	2|27|36|37|37	4	0|66|**123**|262|910	18,347	27,577	4		100%

*Shown are the quality of the reads, number of contigs, read coverage depth, lengths of the consensus genome sequences, and the percent genome coverage (fraction of expected full genome sequence vis-à-vis the consensus scaffold)*.

a *Number represent the minimum | lower quartile | median | upper quartile | maximum depth per position of the sequences*.

b *Numbers of paired-end reads that were used to re-call the consensus sequences*.

c *The genome sequence lengths excludes the poly A tails. Sequences that contained poly(A) tails are in bold*.

d *Number of nt missing at the 5'- and 3'-ends of the consensus sequences vis-à-vis the consensus scaffold*.

e *Percent genome coverage (fraction of expected full genome sequence vis-à-vis the consensus scaffold)*.

### Sequence Analyses

#### Genomic Organization and Features

All 33 complete sequences, with lengths ranging from 27,022 to 27,805 nt excluding poly(A) tails, contain the six IBV genes flanked by 5′- and 3′-UTRs (294–643 nt and 136–446 nt in length, respectively); 14 of the sequences, including the 3 vaccine sequences, have poly(A) tails of variable lengths (Supplementary Table 2). The genomes are organized as 5'UTR-[Rep1a-Rep1b-S-3a-3b-E-M-4b-4c-5a-5b-N-6b]-3'UTR, with 25 sequences having a cassette of seven “accessory” genes (3a, 3b, 4b, 4c, 5a, 5b, and 6b) interspersed variably downstream of gene 2 (S) genomic region. Genes 4b, 4c, and 6b are absent in eight sequences as follows: both genes 4b and 4c are absent in field sequence 2360/20 from Southern Mexico, 4b is absent in sequences 2359/20 and 2754/21 (from Southern Mexico) and 2723/21 (from Northern Mexico), and 6b is absent in the Mass-type vaccine strain 1616/19 and Mass-type/Conn recombinant vaccine strain 1623/19 sequences, and the field sequences 2523/21 and 2598/21 from Central and Northern Mexico, respectively.

##### Gene 1 (Rep1a/1ab Complex)

Lengths of gene 1 (containing 2 overlapping ORFs encoding Rep1a and Rep1ab) varies from 19,490 to 19,970 nt among the 33 sequences, but all sequences have a 4-nt overlap between Rep1a and 1b (Table 2). Whereas, the lengths of Rep1a vary (11,520–11,937 nt), 30 Rep1b sequences are 8,037 nt long; the field sequences 2721/21, 2353/20, and 2752/21 have comparatively shorter Rep1b lengths (7,935, 7,938, and 7,974 nt, respectively). Domain features and borders of accessory genes 2-16 produced from the cleavage of Rep1ab by the virally-encoded proteases PL ^pro^ (gene 3) and 3CL ^pro^ (gene 5) are shown in the Supplementary Table 3. As expected for CoVs (11, 57–60), cleavage sites and lengths of the accessory genes in Rep1a/1ab are conserved across all the sequences, including amino acid residues Q/S required by the 3CL ^pro^ for the cleavage of Rep1ab into Rep1a and 1ab, which releases products of genes10 (exonuclease; 145 amino acids), 11 (unknown function; 23 amino acids), and 12 (RdRp; 917 amino acids). Although the cleavage sites of PL ^pro^ are conserved in all sequenced, their length varies (1,529–1,619 amino acids) compared to the consistent lengths of the main CoV protease, 3CL ^pro^ (307 amino acid residues).

**Table 2:** Nucleotide overlaps between genes and ORFs in the 33 IBV sequences analyzed in this study.

**Sequence**	**Gene 1**	**Genes 1 & 2**	**Genes 2 & 3**	**Gene 3**		**Genes 3 & 4**	**Gene 4**		**Genes 4 & 5**	**Gene 5**	**Genes 5 & 6**	**Gene 6**
	**Rep1a & 1b**			**3a & 3b**	**3b & E**		**M & 4b**	**4b & 4c**		**5a & 5b**		**N & 6b**
Live Mass-type vaccine strain/1616/19	4	50	(VncRNA; 26)	1	20	29	0	80	17	4	58	N/A
4/91 vaccine variant/1619/19	4	50	(VncRNA; 26)	1	20	8	0	80	17	4	58	(VncRNA; 8)
Mass-type-Conn recombinant vaccine strain/1623/19	4	50	1	1	20	29	0	80	17	4	58	N/A
ck/MEX/2353/20	4	50	1	1	17	20	0	80	17	4	58	(VncRNA; 8)
ck/MEX/2354/20	4	50	1	1	20	20	0	80	17	4	58	(VncRNA; 8)
ck/MEX/2359/20	4	50	1	1	20	23	N/A	N/A	17	4	58	(VncRNA; 8)
ck/MEX/2360/20	4	50	1	1	20	23	N/A	N/A	N/A	4	58	(VncRNA; 8)
ck/MEX/2523/21	4	50	1	1	20	23	0	80	17	4	58	N/A
ck/MEX/2562/21	4	50	1	1	20	23	0	80	17	4	58	(VncRNA; 8)
ck/MEX/2563/21	4	50	1	1	17	23	0	80	17	4	58	(VncRNA; 23)
ck/MEX/2592/21	4	50	1	1	20	20	0	80	17	4	58	(VncRNA; 8)
ck/MEX/2598/21	4	50	(VncRNA; 26)	1	20	29	0	80	17	4	58	N/A
ck/MEX/2602/21	4	50	1	1	20	29	0	80	17	4	58	(VncRNA; 23)
ck/MEX/2721/21	4	50	1	1	20	20	(VncRNA; 24)	80	17	4	58	(VncRNA; 23)
ck/MEX/2723/21	4	50	1	1	20	20	N/A	N/A	17	4	58	(VncRNA; 23)
ck/MEX/2725/21	4	50	1	1	20	20	0	80	17	4	58	(VncRNA; 27)
ck/MEX/2731/21	4	50	1	1	20	23	(VncRNA; 13)	17	17	4	58	(VncRNA; 8)
ck/MEX/2742/21	4	50	1	1	20	23	0	80	17	4	58	(VncRNA; 8)
ck/MEX/2743/21	4	50	1	1	38	29	0	95	17	4	58	(VncRNA; 23)
ck/MEX/2748/21	4	50	1	1	17	29	0	80	17	4	58	(VncRNA; 23)
ck/MEX/2752/21	4	50	1	1	17	20	0	80	17	4	58	(VncRNA; 8)
ck/MEX/2753/21	4	50	1	1	17	20	0	80	17	4	58	(VncRNA; 8)
ck/MEX/2754/21	4	50	1	1	17	29	N/A	N/A	17	4	58	(VncRNA; 23)
ck/MEX/2818/21	4	50	1	1	20	20	0	80	17	4	58	(VncRNA; 23)
ck/MEX/2819/21	4	50	1	1	20	20	0	80	17	4	58	(VncRNA; 23)
ck/MEX/2826/21	4	50	1	1	20	20	0	80	17	4	58	(VncRNA; 8)
ck/MEX/2833/21	4	50	1	1	20	20	0	80	17	4	58	(VncRNA; 8)
ck/MEX/2860/21	4	50	1	1	20	29	0	80	17	4	58	(VncRNA; 23)
ck/MEX/2930/21	4	50	1	1	20	(VncRNA; 16)	0	80	17	4	58	(VncRNA; 23)
ck/MEX/2944/21	4	50	1	1	20	4	0	80	17	4	58	(VncRNA; 8)
ck/MEX/2956/21	4	50	1	1	20	23	0	80	17	4	58	(VncRNA; 8)
ck/MEX/2960/21	4	50	1	1	20	23	0	80	17	4	58	(VncRNA; 17)
ck/MEX/2961/21	4	50	1	1	20	29	0	80	17	4	58	(VncRNA; 8)

*The numbers in brackets refer to nt in the VncRNAs*.

##### Gene 2 (S)

**Figure 1:**
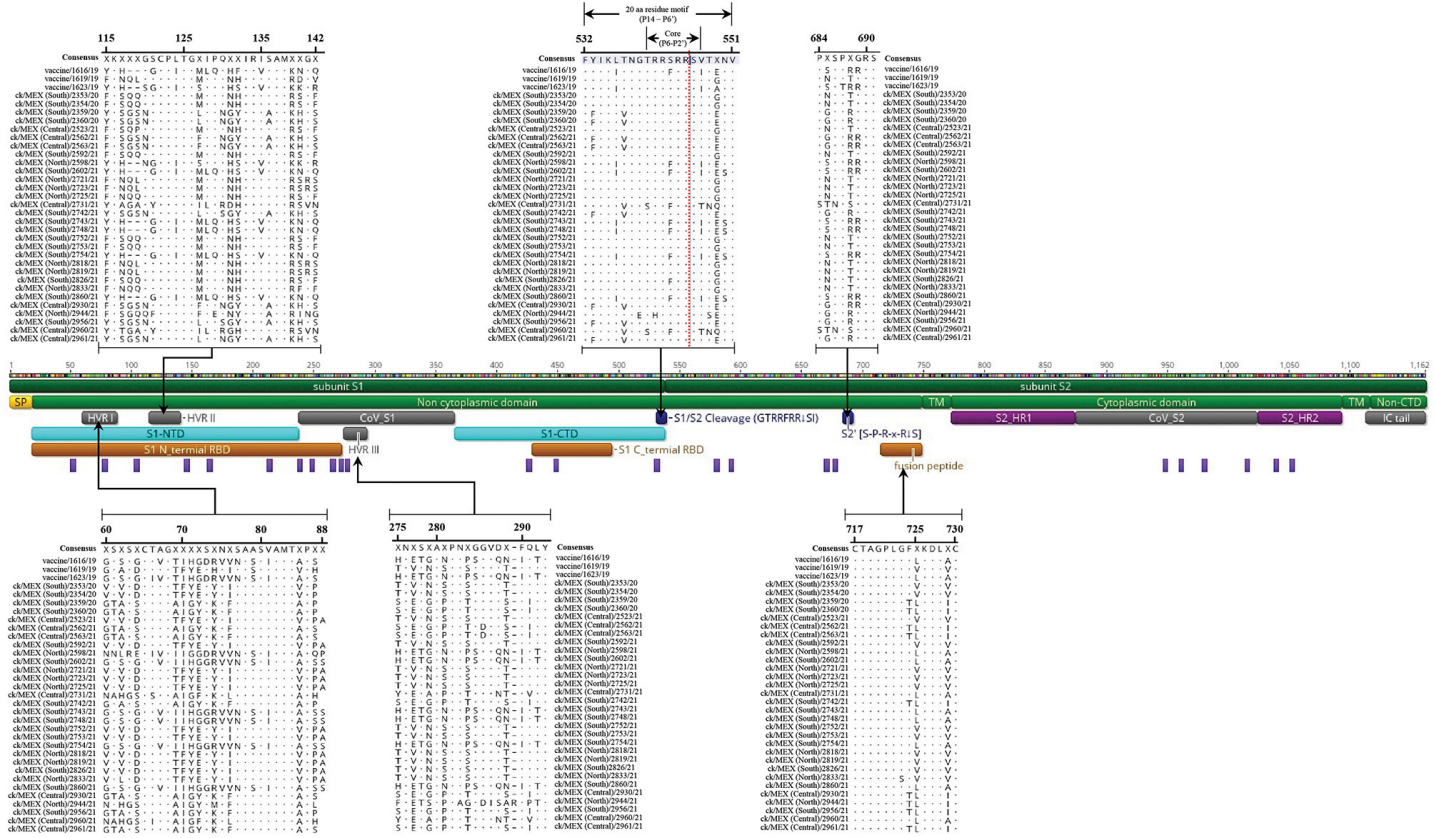
Schematic and amino acid alignment of the overall features of the S glycoprotein. The scheme was constructed in Geneious Prime based on the S protein sequence of the field sequence 2602/21; all 33 S protein sequences in this study have similar general features (see Supplementary Table 4). Sequence IDs are shown on the alignments; the positions indicated at the top of the sequence alignments are in reference to ungapped amino acid residues; dots and dashes indicate identical and deleted amino acid residues, respectively. Features of subunit S1 include N-terminal signal peptide (SP), N-and C-terminal domains (S1-NTD/CTD), which harbor the receptor-binding domains (RBD), and hypervariable regions (HVR I-III). Shown is a 20- residue (running from positions P14–P6') motif furin S1/S2 cleavage site, consisting of a core region (8-residues; position P6–P2'), which harbors the canonical 4-residue motif (**R**x[**K/R]-R**↓**S**); the core region is flanked by two solvent accessible regions (8-residue; P7 to P14, and a 4-residue; P6 to P2'). Note the backward and forward numbering of P1-P14 and P1'-P6', respectively, starting at the conserved R immediately upstream of the cleavage S1/S2 site. The auxiliary S2′ (**P**S(G)S**PR**x**R**↓**S**) cleavage site positioned 153-residues downstream the primary S1/S2 cleavage site is also shown. Subunit S2 domains include fusion peptide (FP), heptad repeat regions (HR1 and HR2), transmembrane domain (TM), and intracellular (IC) tail. Purple vertical bars represent predicted N-linked glycosylation sites.

In all 33 sequences, gene 1 (Rep1a/1ab) and gene 2 (S) overlap by 50 nt (Table 2). Lengths of gene 2 (with a single ORF encoding S glycoprotein) vary, with the most sequences (*n* = 13) having 3,495 nt and others with either 3,510 nt (*n* = 7) or 3,498 nt (*n* = 6) (Supplementary Table 4). The shortest *S* genes are found in the Mass-type vaccine strain 1616/19 (3,462 nt) and the 4/91 vaccine variant 1619/19 (3,468 nt) sequences, and the field sequence 2598/21 (3,453 nt). Whereas, the lengths of subunit *S1* vary (1,602–1,632 nt), all subunit *S2* sequences have lengths of 1,878 nt, except the above-mentioned 3 sequences (1,851-nt long). Figure 1 illustrates general S glycoprotein features. The lengths of HVR I, II and III (residues 60–88, 115–142, and 275–293, respectively) vary among the 33 sequences; HVR I of 27 or 28 residues (in 11 and 22 sequences, respectively), HVR II of 25 or 27 residues (in 8 and 25 sequences, respectively), and HVR III of 18 residues (in all sequences).

All 33 *S*-gene sequences contain the canonical 4 amino acid (aa) residue furin recognition consensus motif **R**-X-[**K**/**R**]-**R**↓**S** at the S1/S2 cleavage site (X is any residue, ↓ is cleavage position and underlined residues are conserved)—Figure 1. The canonical motif is within a 20 aa region (positions P14 to P6', with backward and forward numbering of P1-P14 and P1'-P6', respectively, starting at the conserved R immediate of the R↓S cleavage position), consisting of a core region (8 aa; P6-P2') flanked by two solvent accessible regions (8 aa P7-P14, and a 4 aa; P6-P2') (61). The S1/S2 cleavage motif contains critical physical properties required for furin cleavage and fusion efficiency (61), including absence of the acidic cysteine residue in the core region (P1-P6), presence of a positively-charged residue at position P4 (R is favored), hydrophilic residues in regions flanking the S1/S2 site (positions P7-P10 and P3′-P6′), small hydrophilic residue at position P1′ (S is preferred) and hydrophobic-aliphatic residue at position P2′ (V is favored). There are however, two exemptions in the 8 aa P6-P2′ region: firstly, nine sequences have the hydrophobic F-residue at P3 (instead of preferred hydrophilic residue), and secondly, sequences 2731/21 and 2960/21 have the hydrophilic T-residue at P2′ (instead of the preferred hydrophobic V or I residues). These exceptions are not unusual as the specific interactions of the residues in P2′ and P3 within the furin cleavage pocket are unclear (61).

An auxiliary S2′ cleavage (motif **P**SxS**PR**x**R**↓**S**) is present in 17 sequences positioned 153 amino acid residues downstream of the primary S1/S2 cleavage site (Supplementary Table 4 and Figure 1). Further, all sequences contain a membrane-fusion peptide (FP) in the conserved region flanked by 2 cysteine (C) residues located immediately downstream of the S2′ cleavage site, with the consensus motif **C**TAGPLGF/(T)XK**DL**X**C** (Figure 1); underlined residues C, D, L, and C are conserved across CoVs (62). Numbers of *N*-linked glycosylation sites (on conserved consensus NXS/T motif) varies among the 33 sequences (22–27 sites), with subunit S1 having more sites (*n* = 12–16) compared to subunit S2 (*n* = 10–12); these numbers are within the range of 19–39 sites reported in CoVs (9).

##### Gene 3 (3a/3b and E)

A one-nt overlap occurs between gene 2 (S) and gene 3 (accessory genes 3a-3b and E) in 30 of the 33 sequences. In the Mass-type vaccine 1616/19 and the 4/91 vaccine variant 1619/19 sequences, and the field sequence 2598/21, there is a 26-nt non-coding region (hereafter abbreviated as VncRNA to refer to genomic region between adjacent genes or ORFs without any ORF) between the two genes (Table 2). Within gene 3, there is a 1-nt overlap between 3a and 3b, while 3b and E overlap by 17, 20, and 38 nt in six, 26 and one sequences, respectively. In all sequences, 3a and 3b are of the same lengths (174 and 195 nt, respectively), but the length of the structural E varies (285–330 nt) among the sequences (Supplementary Table 2). Differences in lengths of E-gene is expected due to its extreme divergence in CoVs (9). The E protein sequences in all sequences contained two conserved cysteine residues at position 45 and 46, which serve as E protein palmitoylation sites (63).

##### Gene 4 (M, 4b/4c)

The overlap between gene 3 and gene 4 (M and accessory genes 4b/4c) varies among the sequences (Table 2). Twelve sequences have a 20 nt overlap and nine sequences each have 23-nt and 29-nt overlaps, while the field sequence 2944/21 and the 4/91 vaccine variant 1619/19 sequence have 4 and 8-nt overlaps between the two genes, respectively. There is a 16-nt VncRNA region between the two genes in sequence 2930/21. Gene 4 ORFs vary in length; 654–690 nt (M gene), 102–327 nt (4b), and 132–171 nt (4c; 25 4c are 171 nt in length). Amongst the 3 gene 4 ORFs, there is no overlap of M and 4b in 27 sequences. Sequences 2721/21 and 2731/21 have VncRNAs of 21 nt and 13 nt between M and 4b, respectively. Sequences 2359/20, 2360/20, 2723/2021, and 2754/21 lack 4b; sequence 2360/20 lacks both 4b and 4c (Supplementary Table 2). In 28 sequences, 4b and 4c overlap by 80 nt; the two 2 accessory genes overlap by 17 and 95 nt in sequences 2731/21 and 2743/21, respectively.

##### Gene 5 (5a and 5b)

In all the sequences, gene 4 and gene 5 (5a-5b) overlap by 17 nt; 5a and 5b are 198 and 249 nt in length, respectively, all overlapping by 4-nt (see Table 2 and Supplementary Table 2). The presence and apparent conservation of 5a and 5b across all the 33 sequences in this study agree with reports that all avian CoVs contain gene 5, whose protein products are postulated to contribute to virus/host interactions (64).

##### Gene 6 (N and 6b)

Genes 5 and 6 (N and 6b) overlap by 58 nt in 29 of the 33 sequences (6b is absent in the Mass-type vaccine 1616/19 and Mass-type/Conn recombinant vaccine 1623/19 sequences, and field sequences 2523/21 and 2598/21) (Table 2). Lengths of VncRNAs between N and 6b varies among the sequences; 8 nt (*n* = 16 sequences), 23 nt (*n* = 11 sequences), 17 nt (in sequence 2960/21), and 27 nt (in sequence 2725/21)—Supplementary Table 2. Whereas, all N gene sequences are of the same length (1,230 nt), the lengths of 6b varies widely from 129 to 321 nt amongst the sequences.

#### Sequence Comparison

##### Relationships Between Vaccine and Field Sequences

Supplementary Table 5 presents the results of comparative pairwise nt sequence identities of vaccine vs. field sequences identified in this study. Amongst the three vaccines, the highest identity (100%) is between gene 3 (3a and 3b) the Mass-type vaccine 1616/19 and Mass-type/Conn recombinant vaccine 1623/19 sequences, and the lowest (77.31%) between subunit *S1*-gene of sequences of the Mass-type/Conn recombinant vaccine 1623/19 and the 4/91 vaccine variant 1619/19.

Comparing the vaccine vs. field sequences, the highest identities (99.43–100%) are between genes 3 and 4, and 5a and 5b of field sequence 2598/21 and their homologs in the vaccine sequences. The lowest identity is between 6b (60%) and the 3'-UTR (64.1%) of the field sequences 2723/21 and 2360/20, respectively, and their homologous genomic regions in the 4/91 vaccine variant 1619/19 sequence. For the *S1*-gene sequence, which is used for IBV classification (18), the highest identity (98.14%) is between field sequence 2860/21 and the Mass-type vaccine 1616/19 sequence, while lowest (75.78%) is between the field sequence 2944/21 and the 4/91 vaccine variant 1619/19 sequence. The two field sequences also showed similar identities in their complete *S*-gene sequences (highest and lowest identities of 98.15 and 80.15%, respectively) to the vaccine sequence.

Overall, the most conserved genomic regions amongst the 33 sequences are Rep1ab (88.5–93.24% nt identity) and 5b (93.98–100% nt identity), while the least conserved region are 6b (60–94.22% nt identity) and 4c (74.27–100% nt identity).

##### Relationships With Other Serotypes

**Figure 2:**
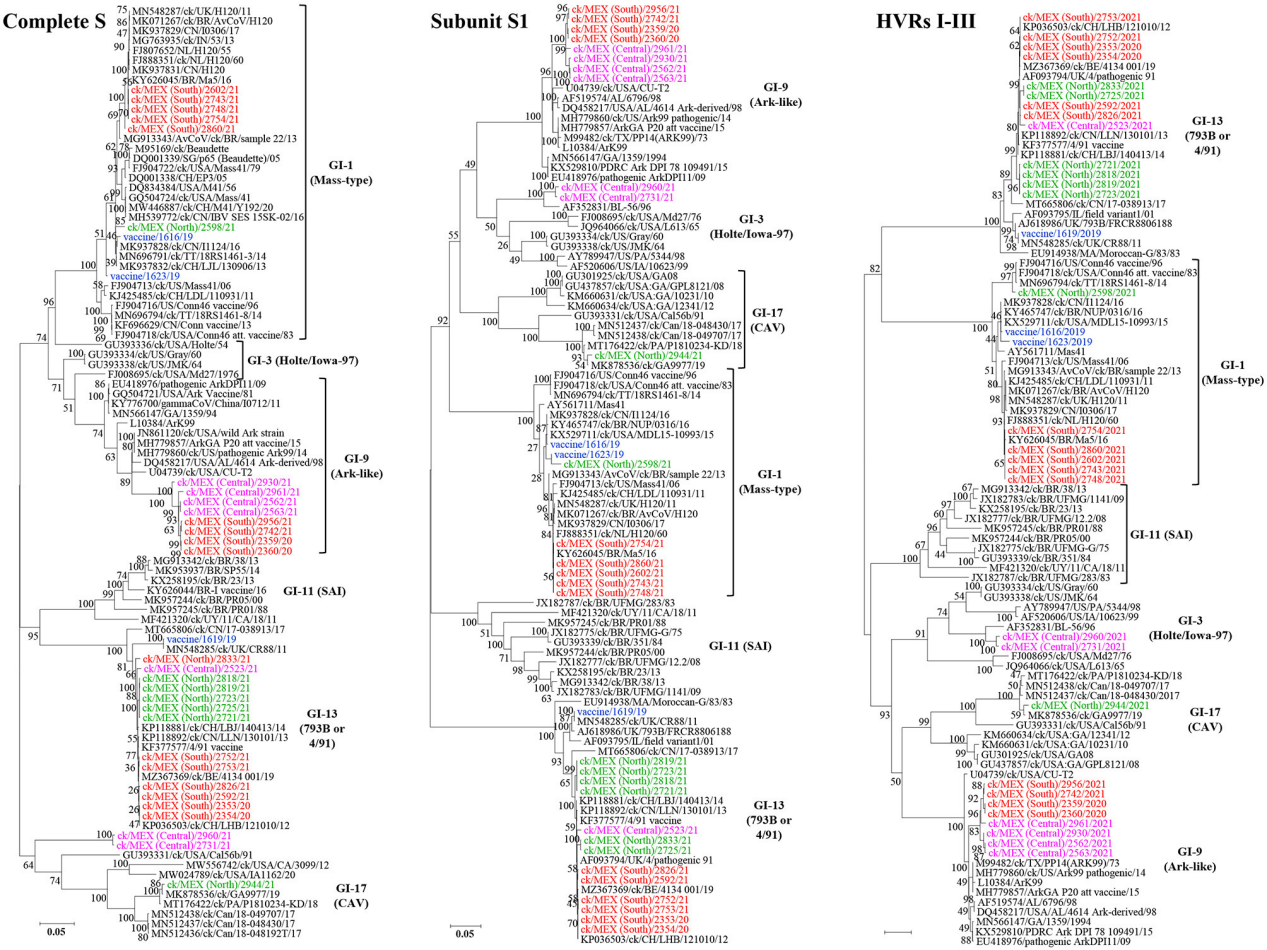
Maximum likelihood phylogenetic tree of nt sequences of subunit *S1*-gene, hypervariable regions I-III (HVRs I-III) of the S1-gene, and complete *S*-gene using GTR model in MEGA 6. The 33 sequences in this study (3 vaccine sequences highlighted in blue; 30 field sequences; color-coded based on sampling regions in Mexico) clustered with serotypes in 5 lineages of IBVs, i.e., lineages GI-1 (Mass-type serotypes; *n* = 8 sequences), GI-3 (Holte/Iowa/97 serotypes; *n* = 2 sequences), GI-9 (Ark-like serotypes; *n* = 8 sequences), GI-13 (793B also known as 4/91 serotypes*; n* = 14 sequences), and GI-17 (CAVs; *n* = 1 sequence). The analysis involved 94 sequences. All positions with <95% site coverage were eliminated. The final datasets had 1,576, 757, and 3,431 positions for the complete S1-gene, HVRs I-III, and complete S-gene, respectively.

Relationships between the 33 sequences in this study with other IBVs are summarized in Table 3 (complete S-gene sequences) and Supplementary Table 6 (complete genome sequence). Phylogenetic trees based on nt sequences of complete *S*-gene, *S1*-gene, and HVRs I-III classified the 33 sequences in this study within five different lineages (Figure 2).

**Table 3:** BLASTn results of the 33 complete *S*-gene sequences in this study.

**Sequence**	**Sampling date**	**Origin (region)**	**Flock age (days/weeks)**	**Tissue**	**Best BLASTn hit (isolate)**	**Lineage (serotype)**	**Seq. length**	**Hit start**	**Hit end**	**Query coverage**	**Bit-Score**	**Identity %**
OM912698/live Mass-type vaccine strain/1616/19	23-Apr-19	n/a	Vaccine	BIAH Mexico	MK937828/ck/CN/I1124/16	GI-1 (Mass-type)	3,462	20,368	23,829	100%	6,558.98	99.51
OM912697/4/91 vaccine variant strain/1619/19	23-Apr-19	n/a	Vaccine	BIAH Mexico	MN548285/ck/UK/CR88/11	GI-13 (793B or 4/91)	3,468	20,371	23,838	100%	6,557.03	99.94
OM912696/Mass-type-Conn recombinant vaccine strain/1623/19	23-Apr-19	n/a	Vaccine	BIAH Mexico	MN696791/ck/TT/18RS1461-3/14	GI-1 (Mass-type)	3,489	1	3,489	100%	6,391.7	98.45
OM912680/ck/MEX/2353/20	25-Nov-20	South	Broiler (42D)	choanal/lung	KP036503/ck/CH/LHB/121010/12	GI-13 (793B or 4/91)	3,495	20,314	23,808	100%	6,720.48	100
OM912682/ck/MEX/2354/20	25-Nov-20	South	Broiler (42D)	spleen/bursa	KP036503/ck/CH/LHB/121010/12	GI-13 (793B or 4/91)	3,495	20,314	23,808	100%	6,720.48	100
OM912678/ck/MEX/2359/20	25-Nov-20	South	Broiler (42D)	choanal/lung	DQ458217/AL/4614/98	GI-9 (Ark)	3,510	1	3,510	100%	5,400.72	94.42
OM912677/ck/MEX/2360/20	25-Nov-20	South	Broiler (42D)	spleen/bursa	DQ458217/AL/4614/98	GI-9 (Ark)	3,510	1	3,510	100%	5,605.32	94.42
OM912695/ck/MEX/2523/21	28-Jan-21	Center	Broiler (29D)	choanal/lung	KP118891/ck/CH/LHLJ/111246/11	GI-13 (793B or 4/91)	3,495	20,314	23,808	100%	6,576.28	99.28
OM912694/ck/MEX/2562/21	25-Feb-21	Center	Broiler (28D)	choanal/lung	DQ458217/AL/4614/98	GI-9 (Ark)	3,510	1	3,510	100%	5,605.32	94.25
OM912693/ck/MEX/2563/21	25-Feb-21	Center	Broiler (28D)	spleen/bursa	DQ458217/AL/4614/98	GI-9 (Ark)	3,510	1	3,510	100%	5,576.48	94.19
OM912692/ck/MEX/2592/21	1-Apr-21	South	Broiler (21D)	choanal/lung	KP036503/ck/CH/LHB/121010/12	GI-13 (793B or 4/91)	3,495	20,314	23,808	100%	6,708.95	99.94
OM912685/ck/MEX/2598/21	1-Apr-21	North	Broiler (27D)	spleen/bursa	MK937828/ck/CN/I1124/16	GI-1 (Mass-type)	3,453	20,368	23,829	100%	6,335.94	98.5
OM912684/ck/MEX/2602/21	1-Apr-21	South	Broiler (21D)	spleen/bursa	KY626045/BR/Ma5/16	GI-1 (Mass-type)	3,489	20,314	23,802	100%	6,685.87	99.89
OM912691/ck/MEX/2721/21	6-Jun-21	North	Broiler (21D)	choanal/lung	KP118880/ck/CH/LHB/130927/13	GI-13 (793B or 4/91)	3,495	20,314	23,808	100%	6,628.19	99.54
OM912690/ck/MEX/2723/21	6-Jun-21	North	Broiler (28D)	choanal/lung	KP118880/ck/CH/LHB/130927/13	GI-13 (793B or 4/91)	3,495	20,314	23,808	100%	6,645.5	99.63
OM912689/ck/MEX/2725/21	6-Jun-21	North	Broiler (21D)	choanal/lung	KP118880/ck/CH/LHB/130927/13	GI-13 (793B or 4/91)	3,495	20,314	23,808	100%	6,383.14	99.63
OM912676/ck/MEX/2731/21	23-Jun-21	Center	Broiler (23D)	choanal/lung	GU393334/ck/US/Gray/60	GI-3 (Holte/Iowa-97)	3,498	20,382	23,874	99.69%	4,052.67	87.66
OM912688/ck/MEX/2742/21	11-Jul-21	South	Broiler (28D)	spleen/bursa	DQ458217/AL/4614/98	GI-9 (Ark)	3,510	1	3,510	100%	5,599.55	94.39
OM912683/ck/MEX/2743/21	11-Jul-21	South	Broiler (28D)	choanal/lung	KY626045/BR/Ma5/16	GI-1 (Mass-type)	3,489	20,314	23,802	100%	6,685.87	99.89
OM912687/ck/MEX/2748/21	15-Jul-21	South	Broiler (28D)	choanal/lung	KY626045/BR/Ma5/16	GI-1 (Mass-type)	3,489	20,314	23,802	100%	6,685.87	99.89
OM912679/ck/MEX/2752/21	20-Jul-21	South	Broiler (21D)	choanal/lung	KP036503/ck/CH/LHB/121010/12	GI-13 (793B or 4/91)	3,495	20,314	23,808	100%	6,708.95	99.94
OM912686/ck/MEX/2753/21	20-Jul-21	South	Broiler (28D)	spleen/bursa	KP036503/ck/CH/LHB/121010/12	GI-13 (793B or 4/91)	3,495	20,314	23,808	100%	6,708.95	99.94
OM912681/ck/MEX/2754/21	20-Jul-21	South	Broiler (21D)	choanal/lung	KY626045/BR/Ma5/16	GI-1 (Mass-type)	3,489	20,314	23,802	100%	6,685.87	99.89
OM912699/ck/MEX/2818/21	9/10/2021	North	Broiler (28D)	choanal/lung	KP118880/ck/CH/LHB/130927/13	GI-13 (793B or 4/91)	3,495	20,314	23,808	100%	6,377.6	99.6
OM912700/ck/MEX/2819/21	9/10/2021	North	Broiler (28D)	spleen/bursa	KP118880/ck/CH/LHB/130927/13	GI-13 (793B or 4/91)	3,495	20,314	23,808	100%	6,377.6	99.6
OM912701/ck/MEX/2826/21	9/13/2021	South	Broiler (28D)	choanal/lung	KP036503/ck/CH/LHB/121010/12	GI-13 (793B or 4/91)	3,495	20,314	23,808	99.99%	6,427	99.86
OM912702/ck/MEX/2833/21	9/27/2021	North	Broiler (21D)	choanal/lung	JN192154/ck/4/91(UK)	GI-13 (793B or 4/91)	3,495	1	3,492	99.91%	6,267	99.1
OM912703/ck/MEX/2860/21	10/12/2021	South	Broiler (28D)	choanal/lung	KY626045/BR/Ma5/16	GI-1 (Mass-type)	3,489	20,314	23,802	100%	50,190	99.46
OM912704/ck/MEX/2930/21	23-Nov-21	Center	Broiler (21D)	choanal/lung	DQ458217/AL/4614/98	GI-9 (Ark)	3,507	1	3,510	99%	5,441.35	94.65
OM912705/ck/MEX/2944/21	7-Dec-21	North	Broiler (28D)	choanal/lung	MK878536/ck/GA9977/19	GI-17 (CAV)	3,501	20,371	23,871	100%	6,377.6	99.54
OM912706/ck/MEX/2956/21	14-Dec-21	South	Broiler (28D)	choanal/lung	DQ458217/AL/4614/98	GI-9 (Ark)	3,510	1	3,510	100%	5,400.72	99.42
OM912707/ck/MEX/2960/21	14-Dec-21	Center	Layer (7.6 W)	spleen/bursa	GU393334/ck/US/Gray/60	GI-3 (Holte/Iowa-97)	3,498	20,382	23,874	100%	4,024.97	87.52
OM912708/ck/MEX/2961/21	15-Dec-21	Center	Broiler (21D)	choanal/lung	DQ458217/AL/4614/98	GI-9 (Ark)	3,510	1	3,510	100%	5,373.02	94.28

*Lineage (serotype) classification is based on S1-gene sequence (18). Genome length excludes poly(A) tails. GenBank accession numbers are shown for the best BLASTn hit for each sequence*.

###### Lineage GI-1 (Mass-Type).

As shown in Table 3, six and two field and vaccine sequences are closely related to three different Mass-type strains, and the Mass-type/Conn recombinant vaccine 1623/19 sequence is 98.45% similar to the pathogenic field strain MN696791/ck/T&T/18RS1461-3/14 isolated from a vaccinated broiler with respiratory disease (65). The Mass-type vaccine 1616/19 sequence and field sequence 2598/21 from Northern Mexico are 99.51 and 98.51% identical to strain MK937828/ck/CN/I1124/16, respectively. The remaining five field sequences from southern Mexico (2602/21, 2754/21, 2743/21, 2748/21, 2860/21), are 99.46–99.89% identical to a Brazilian government-licensed Ma5 vaccine strain [KY626045/BR/Ma5/16; (66)]. Phylogenetic clustering based on complete *S*-gene, *S1*-gene and HVRs I-III mirrored the BLASTn results, where the above-mentioned five sequences segregate in a subclade containing the Brazilian Ma5 and Canadian/European/Brazilian H120 vaccine strains (Figure 2). Based on the HVR I-III, the Mass-type vaccine 1616/19 and Mass-type/Conn recombinant vaccine 1623/19 sequences are in a subclade containing the 2016 Chinese strain and a representative of Mass-type viruses (i.e., AY561711/Mas41), while the field sequence 2598/21 is in a distinct subclade containing the T&T and Connecticut vaccine strains. All of the 8 Mass-type sequences in this study contained an S2′ site downstream of the primary S1/S2 cleavage site (Supplementary Table 4 and Figure 1).

###### Lineage GI-3 (Holte/Iowa-97).

Based on complete *S*-gene sequences, viruses 2960/21 and 2731/21 from Central Mexico match closest (87.52 and 87.66% nt identities) to strain GU393334/ck/US/Gray/60 belonging to lineage GI-3, which comprises of respirotropic and nephropathogenic Holte, JMK, Gray and Iowa-97 viruses (18, 67). However, BLASTn searches using complete genome sequences returned closest hit (93.39 and 92.99%) to the pathogenic field strain MH779860/ck/USA/Ark99/14 of lineage GI-9 (Supplementary Table 6). From Figure 2, complete *S*-gene does not phylogenetically place the two sequences with serotypes in either lineages GI-3 or GI-9; rather, the sequences are in a distinct subclade within a larger clade containing California variants (CAVs; lineage GI-17). However, based on the *S1*-gene and HVRs I-III, these sequences cluster with lineage GI-3 serotypes, but in a distinct subclade containing strain AF352831/BL-56/96, which has been previously described as uniquely Mexican (25).

###### Lineage GI-9 (Ark-Like).

The North American lineage GI-9 serotypes were implicated in the rolling reactions in vaccinated flocks and the persistence of respiratory syndromes in flocks (25). Based on the complete *S*-gene sequences (Table 4), four sequences from Central Mexico (2930/21, 2961/21, 2562/21, and 2563/21), and four from Southern Mexico (2742/21, 2956/21, 2359/20, and 2360/20), are all closely-related to DQ458217/US/AL/4614/98 (94.19–94.65% nt identities), an Ark-DPI-derived vaccine virus originally isolated from a 40-day-old chicken with respiratory disease (68). Complete genome sequences returned similar hits, except that seven sequences are closest to an Ark pathogenic field strain (94.07–99.82% nt identity), while an attenuated ArkGA vaccine virus (MH779857/ck/USA/ArkGA-P20/15) is the closest match to sequence 2961/21 with 93.7% nt identity (Supplementary Table 6). Based on complete *S*-/*S1*-gene and HVRs I-III, all 8 sequences phylogenetically cluster in separate subclade distinct from other Ark-like strains, supported by bootstrap values of 100% (*S*-/*S-1* genes) and 83% (HVRs I-III). As in the case of Mass-type viruses, all 8 Ark-like sequences contained the S2′ cleavage (Supplementary Table 4 and Figure 1).

**Table 4:** Recombination events in the complete *S*-gene sequences in this study.

**Potential recombinant seq**		**Breakpoints**			**“Major parent” sequence (nt)** ^**a**^		**“Minor parent” sequence (nt)** ^**b**^		**Confirmation of recombination event**	
**Sequence**	**Lineage**	**Start -> end (nt length)**	**Start -> end (aa length)**	**Domain in aa seq**	**Sequence**	**Lineage**	**Sequence**	**Lineage**	**Detection algorithm^c^**	**Corrected av. *p*-value**
OM912707/ck/MEX (Central)/2960/21	GI-3 (Holte/Iowa-97)	2,965 -> 3,355 (391)	989 -> 1,118 (130)	HR2	FJ904713/ck/US/Mass41/06 (84%)	GI-1 (Mass-type)	OM912693/ck/MEX (Central)/2563/21 (99.5%)	GI-9 (Ark-like)	GENECONV (2), BootScan (2), MaxChi (2), Chimaera (2), SiSscan (2), 3Seq (2)	8.115 E-18
OM912676/ck/MEX (Central)/2731/21	GI-3 (Holte/Iowa-97)	2,965 -> 3,355 (391)	989 -> 1,118 (130)	HR2	FJ904713/ck/US/Mass41/06 (84%)	GI-1 (Mass-type)	OM912693/ck/MEX (Central)/2563/21 (99.1%)	GI-9 (Ark-like)	RDP (1), GENECONV (2), BootScan (2), MaxChi (2), Chimaera (2), SiSscan (2), 3Seq (2)	5.934 E-07
OM912696/Mass-type-Conn recombinant vaccine strain/1623/19	GI-1 (Mass-type)	1,868 -> 2,550 (683)	624 -> 850 (227)	FP – HR1	DQ834384/USA/M41/56 (98%)	GI-1 (Mass-type)	FJ904716/US/Conn46 vaccine/96 (99.1%)	GI-1 (Mass-type)	GENECONV (1), BootScan (1), MaxChi (1), Chimaera (1), SiSscan (1), 3Seq (1)	2.527 E-10
OM912693/ck/MEX (Central)/2563/21	GI-9 (Ark-like)	1,710 -> 2,395 (686)	571 -> 798 (228)	FP – HR1	Unknown^*^ (GU393331/ck/USA/Cal56b/91)	GI-17 (CAV)	MN512438/ck/Can/18-049707/17 (97.2%)	GI-17 (CAV)	GENECONV (7), BootScan (7), MaxChi (7), Chimaera (7), SiSscan (7), 3Seq (4)	5.19 E-26
OM912703/ck/MEX (South)/2860/21	GI-1 (Mass-type)	1,749 -> 2,272 (524)	584 -> 757 (174)	FP	FJ888351/ck/NL/H120/60 (99.8%)	GI-1 (Mass-type)	MK937828/ck/CN/I1124/16 (100%)	GI-1 (Mass-type)	GENECONV (1), BootScan (1), MaxChi (1), Chimaera (1), SiSscan (1), 3Seq (1)	4.509 E-06
OM912704/ck/MEX (Central)/2930/21	GI-9 (Ark-like)	1,707 -> 2,392 (686)	570 -> 797 (228)	FP – HR1	Unknown^*^ (GU393331/ck/USA/Cal56b/91)	GI-17 (CAV)	MN512438/ck/Can/18-049707/17 (93.7%)	GI-17 (CAV)	GENECONV (7), BootScan (7), MaxChi (7), Chimaera (7), SiSscan (7), 3Seq (4)	1284 E-15
OM912705/ck/MEX (North)/2944/21	GI-17 (CAV)	1,701 -> 2,386 (686)	568 -> 795 (228)	FP – HR1	Unknown^*^ (GU393331/ck/USA/Cal56b/91)	GI-17 (CAV)	MN512438/ck/Can/18-049707/17 (95.5%)	GI-17 (CAV)	GENECONV (7), BootScan (7), MaxChi (7), Chimaera (7), SiSscan (7), 3Seq (4)	2.284 E-19
OM912697/4/91 vaccine variant strain/1619/19	GI-13 (793B or 4/91)	2,786 -> 3,156 (371)	930 -> 1,052 (123)	HR2	ck/MEX (North)/2833/21 (96.8%)	GI-13 (793B or 4/91)	Unknown^*^ (FJ904716/US/Conn46 vaccine/96)	GI-1 (Mass-type)	RDP (1), GENECONV (1), BootScan (2), MaxChi (2), Chimaera (2), 3Seq (2)	7.266 E-13
OM912685/ck/MEX (North)/2598/21	GI-1 (Mass-type)	56 -> 681 (626)	20 -> 227 (208)	RBD (HVR-I – HVR-II)	MK937828/ck/CN/I1124/16 (99.8%)	GI-1 (Mass-type)	FJ904716/US/Conn46 vaccine/96 (99.8%)	GI-1 (Mass-type)	GENECONV (1), BootScan (1), MaxChi (1), Chimaera (1), SiSscan (1), 3Seq (1)	7.466 E-27

**Figure 3:**
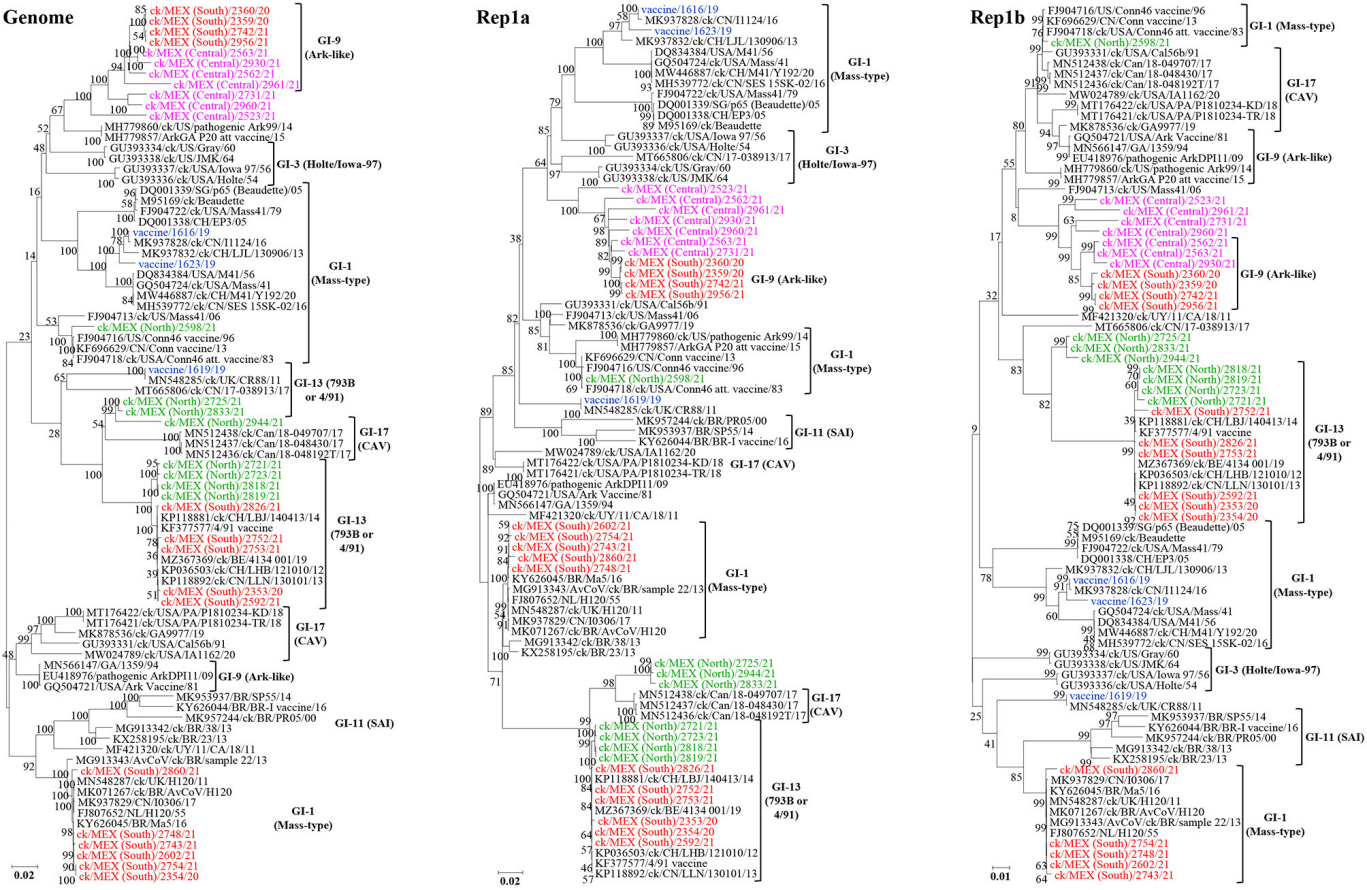
Maximum likelihood phylogenetic tree nt sequences of complete genome, Rep1a, and Rep1b using GTR model in MEGA 6. The 3 vaccine sequences are highlighted in blue color; the 30 field sequences are color-coded based on sampling regions in Mexico. The analysis involved 83 sequences. All positions with <95% site coverage were eliminated. The final dataset had 26,895 (complete genome), 11,782 (Rep1a), and 8,037 (Rep1b) positions.

*The analysis, which involved a total of 70 nucleotide sequences, was performed using RDP, GENECONV, MaxChi, BootScan, SiSscan, 3Seq, and Chimaera programs executed in the RDP v4.101 suite (54). The nt identities between the transferred fragment in the recombinant and the major/minor parent sequences are indicated. Phylogenetic relationships of the potential recombinants (based on alignment of the regions derived from the major parents), the major/minor parents and other viruses are presented in Figure 3. GenBank accession numbers are shown for each sequence*.

a *“Major parent” indicates sequence in other viruses most closely related to the sequence surrounding the transferred fragment*.

b *“Minor parent” indicates the sequence closely related to the fragment in the recombinant*.

c *For each detection algorithm, the number of sequences in which the transferred fragment was detected is shown in brackets*.

* *“Unknown” indicates that only one parent and a recombinant need be in the alignment for transferred fragment to be detectable (sequence in bracket was used to infer existence of the unknown parent)*.

*FP, membrane-fusion peptide; HR, heptad repeat region; HVR, hypervariable region; RBD, receptor-binding domains*.

###### Lineage GI-13 (793B, 4/91 or CR88).

Genome sequences of 13 field samples and the 4/91 vaccine variant 1619/19 match closest to and cluster with lineage GI-13 serotypes (see Table 4, Supplementary Table 6, and Figure 2). Although field and vaccine 793B-like sequences have a near global presence, they have rarely been reported in Latin America. Both complete genome and S-gene of the 4/91 vaccine variant 1619/19 is closest MN548285/ck/UK/CR88/11 (99.94% and 99.98% nt identities, respectively). The 13 field sequences match to different strains depending on sampling regions (Table 4). Sequences 2353/20, 2354/20, 2592/21, 2752/21, 2753/21, and 2826/21 from Southern Mexico are 99.86–100% identical to KP036503/ck/CH/LHB/121010/12 based on complete *S*-gene sequences, while complete genome sequences are 99.87–99.97% identical to isolate MZ367369/ck/Belgium/4134 001/19. Six of the seven sequences from Northern Mexico (2721/21, 2723/21, 2725/21, 2818/21, and 2819/21) have nt identities of 99.54–99.63% to isolate KP118880/ck/CH/LHB/130927/13, while the field sequence 2833/21 is 99.1% identical to isolate JN192154/ck/4/91. Sequence 2523/21, from Central Mexico, is 99.28% similar to strain KP118891/ck/CH/LHLJ/111246/11 based on the complete S-gene, but the complete genome sequence is 92.96% identical MT665806/ck/Canada/17-038913/17. The BLASTn results are reflected in the phylogenetic clustering based on complete *S*-gene, *S1*-gene and the HVRs I-III, where the vaccine sequence group with CR88 strain is in a distinct subclade away from the field sequences (Figure 2).

###### Lineage GI-17 (California Variants; CAVs).

Complete *S*-gene sequence of the field sequence 2944/21 from Northern Mexico is 99.54% identical to a Delmarva (DMV) virus isolated from broiler chickens [MK878536/ck/GA9977/19; (69)]—Table 4. The complete genome sequence is 96.04% identical to strain MN512437/ck/Can/18-048430/17. Phylogenetically, the *S*-/*S1*-gene trees group the Mexican field sequence with strains GA9977 and MT176422/ck/PA/P1810234-KD/18 in a subcluster distinct from other Canadian and USA viruses, but the HVRs I-III tree does not include the P1810234-KD strain (Figure 2). Again, like in the Mass-type and Ark-like viruses, the CAV-like sequence 2944/21 contained S2′ cleavage (Supplementary Table 4 and Figure 1).

We also performed phylogenetic analyses based on complete genome and Rep1ab (Figure 3), structural genes E, M and N (Supplementary Figure 1), and 3ab, 5a/b, and 6b (Supplementary Figure 2). Notably, none of the 33 sequences cluster with lineage GI-11 (SAI serotypes), which have been described as serologically and phylogenetically unique to South America (29–31). However, the previously described Mexican SAI viruses group in distinct subclades within larger clades containing Mass-type (based on nt sequences of the complete genome, Rep1b and 3a/b) and 793B (based on nt sequences of the complete *S*-gene, structural genes E/M and 5a/b) strains.

##### Indels and Mutations in the HVRs of S1-Gene Sequence

**Figure 4:**
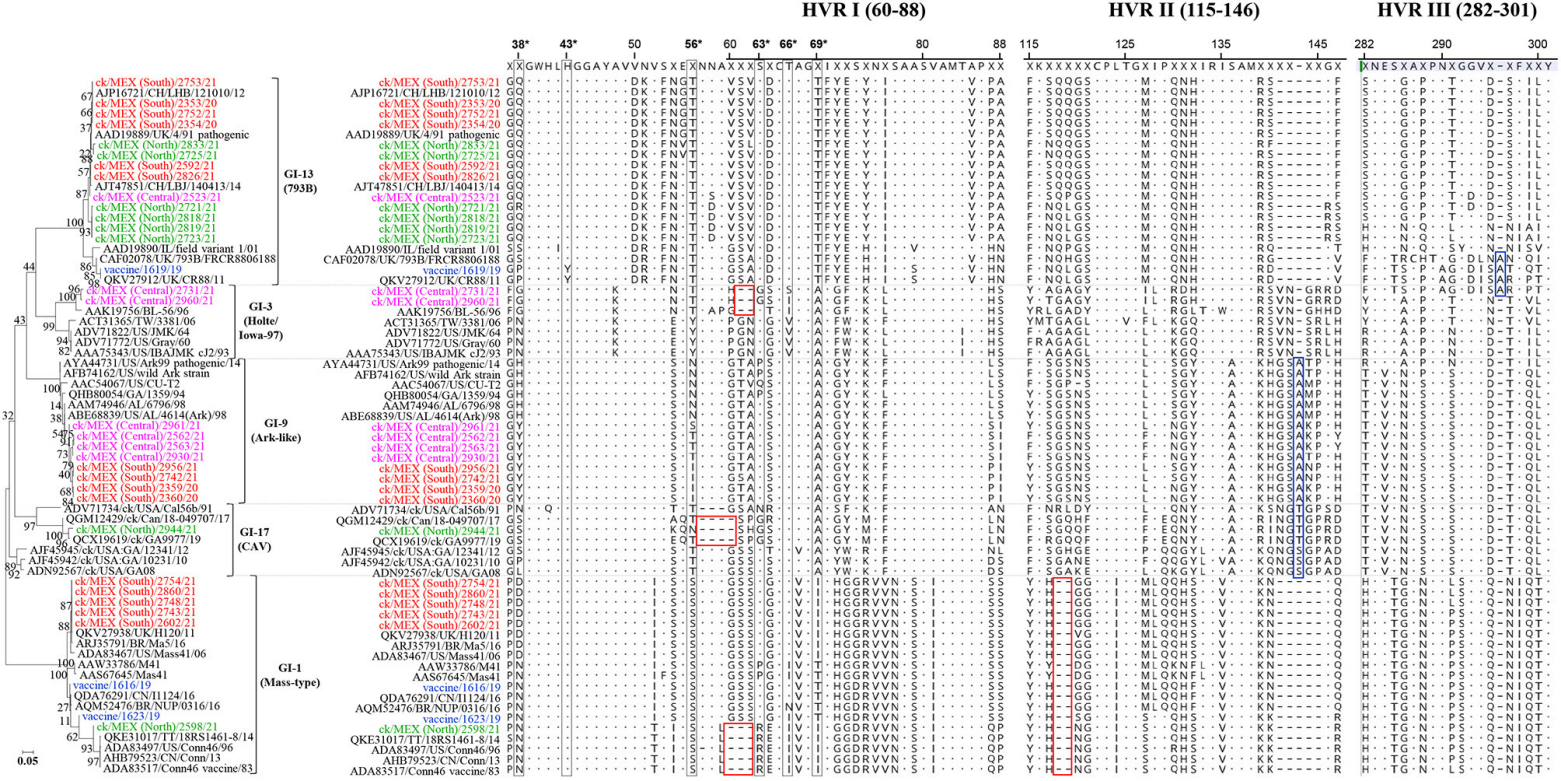
Analyses of the *S1* glycoprotein HVRs I- III (amino acid residues 37-301). The phylogenetic tree was constructed using the JTT matrix-based model in MEGA and involved 67 sequences and a final dataset consisting of 253 positions. The 33 sequences in this study (3 vaccine sequences highlighted in blue; 30 field sequences; color-coded based on sampling regions in Mexico) assembled in this study are aligned with representatives of serotypes belonging to 5 IBV lineages. Dots and dashes in the alignments indicate identical and deleted amino acid residues, respectively. Amino acid residues critical for attachment of the Mass-type prototype (M41; GenBank accession No. AY851295) to chicken respiratory tract tissues are boxed in black [i.e., N38, H43, S56, P63, I66, and T69 (14)]; red and blue boxes indicate deleted and inserted amino acid residues, respectively.

Alignment of translated S1 HVR I (residues 37–88), HVR II (residues 115–146) and HVR III (residues 282–301) sequences revealed considerable variations among the 33 IBVs (Figure 4).

###### HVR I.

Six residues in HVR I of the Mass-type prototype AY851295/M41 (i.e., N38, H43, S56, P63, I66, and T69) are critical for viral attachment to respiratory tract tissues (14). Within the Mass-type sequences, field sequence 2598/21 from Northern Mexico, and the Mass-type vaccine 1616/19 and Mass-type/Conn recombinant vaccine 1623/19 sequences, have an asparagine in position 38 (N38) similar to the M41 sequence, while all five Mass-type sequences from Southern Mexico have an aspartic acid at position 38 (D38) similar to the Brazilian Ma5 vaccine, UK H120 and 2006 Mass41 sequences. The consensus histidine at position 43 (H43) is conserved across all 33 sequences, except in the 4/91 vaccine variant 1619/19 sequence (lineage GI-13), which has a substitution of histidine with tyrosine (H43Y) similar to the CR88 strain from UK. Serine and threonine residues at positions 56 (S56 and T56) are conserved amongst the 793B-like and Mass-type sequences, respectively, but varies among the Ark-like sequences. Further, the critical amino acid at position 63 (S63) is conserved in the sequences across the five IBV lineages used in the alignment, except in field sequences 2731/21 and 2960/21 (from Central Mexico) and sequence 2944/21 (from Northern Mexico), which have S63G substitution, and sequence 2598/21 (from Northern Mexico) with an S63R substitution. Position 66 is largely conserved across sequences in all 5 lineages, while position 69 appear to be lineage-specifically conserved, except in the Mass-type vaccine 1616/19 and Mass-type/Conn recombinant vaccine 1623/19 sequences, which have T69 compared the six field sequences from Southern (*n* = 5) and Northern (*n* = 1) Mexico in the Mass-type group.

In addition to substitutions, HVR I has three instances of deletion. One is in field sequences 2731/21 and 2960/21 from Central Mexico (in lineage GI-3) with a 2-residue deletion (positions 61–62), which is also present in the Mexican BL-56 strain. Another instance is a 4-residue deletion (positions 57–60) in the field sequence 2944/21 from Northern Mexico (lineage GI-17), which is also present in the DMV strains MK878536/ck/GA9977/19 and MN512438/ck/Can/18-049707/17. The third is a 3-residue deletion (positions 60 to 62) in field Mass-type sequence 2598/21 from Northern Mexico, which is present in strain QKE31017/T&T/18RS1461-8/14) and three Conn vaccine strains from USA.

###### HVR II.

Most amino acid variations in HVR II are between the positions 116–121 and 138–147 (Figure 4). For example, in lineage GI-13, all the six field sequences from Northern Mexico, and the 4/91 vaccine variant 1619/19 sequence have N117 (similar to CR88 strains from UK and a field variant from Ireland), while all six field sequences from Southern Mexico have S117 (similarly present in a pathogenic 4/91 strain from UK and two other Chinese strains). The Mass-type vaccine 1616/19 and Mass-type/Conn recombinant vaccine 1623/19 sequences, and all the field sequences in lineage GI-1 (one sequence from northern and five from Southern Mexico) have a 2-residue deletion between positions 117 and 120. Additionally, some of the field sequences from Northern (*n* = 5) and Central (*n* = 2) Mexico have a G145R substitution, also present in the Canadian and USA DMV-like strains. All eight field Ark-like sequences (four each from Central and Southern Mexico), and the CAV-like sequence 2944/21 from Northern Mexico have a single residue insertion between positions 142 and 143.

###### HVR III.

One of the variations in the HVR III is in the Ark-like sequences, where all eight field sequences have E284V substitution compared to the USA pathogenic field strain AYA44731/US/Ark99. This substitution is also present in the CAV viruses, including in field sequence 2944/21 from Northern Mexico. Another variation is in the 4/91 vaccine variant 1619/19 sequence and the Holte/Iowa-97-like field sequence 2731/21 from Central Mexico, which have a single amino acid (alanine) insertion at position 296, which is also present in the CR88 strains AJ618986/UK/FR-88061-88 and MN548285/ck/UK/CR88/11.

### Recombination Events

**Figure 5:**
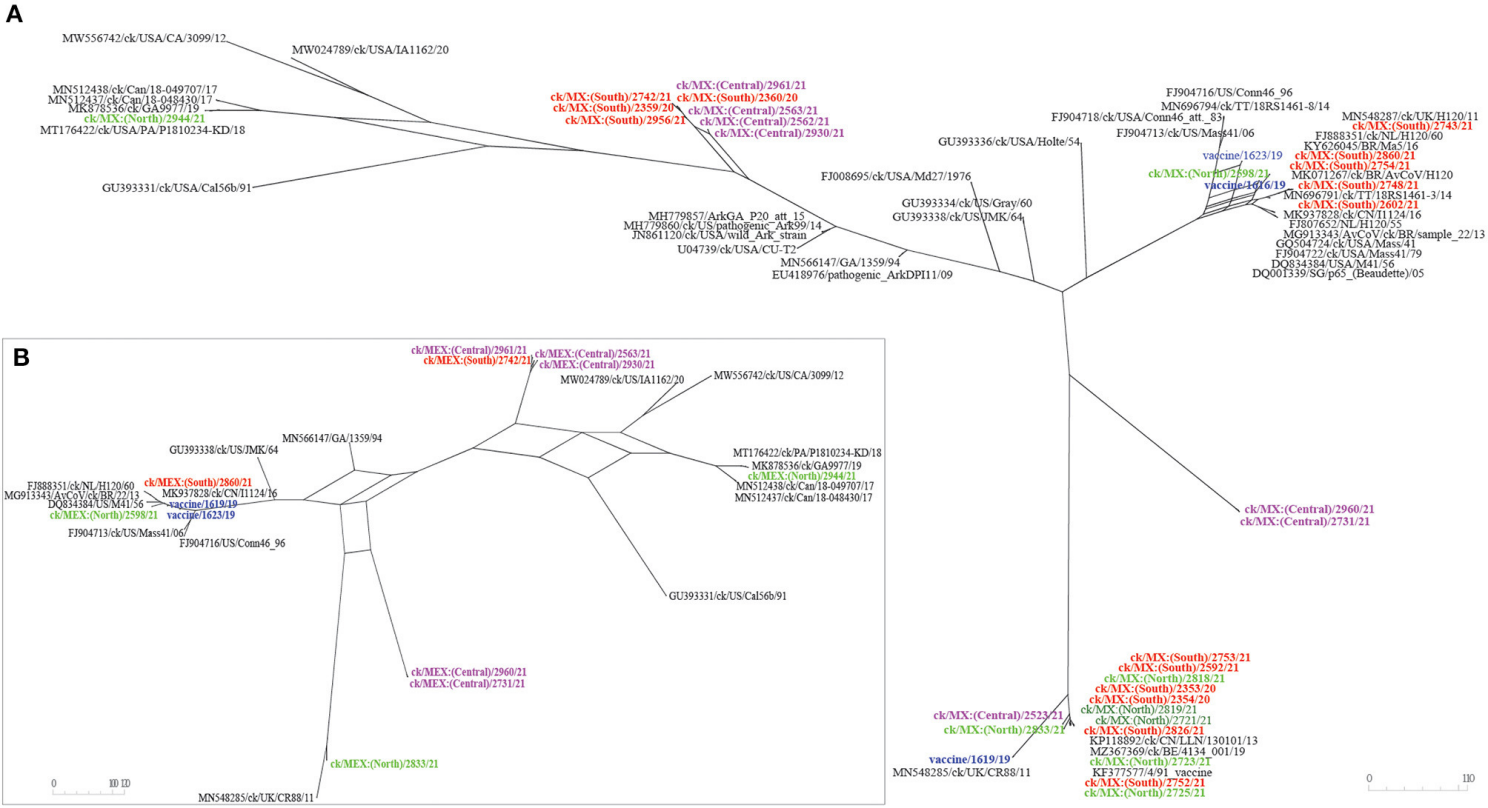
Reticulate network using complete *S*-gene sequences constructed using SplitsTree5 v 5.0.0 (53). The network predicts putative evolutionary histories of the IBVs, where the internal nodes and the edges correspond to ancestral taxa and patterns of descents, respectively. Nodes with more than two parents represent reticulate events (e.g., recombination, horizontal gene transfer, or hybridization). “Split” is a partition of the taxa into two subsets with the edges separating the taxa subsets of the split from those on the other side of the split (the length of the edge in the network is proportional to the weight of the split it is associated with, which is analogous to branches in conventional phylogenetic trees). **(A)** The input contained 70 taxa and 101 trees constructed using Splits Network Algorithm with default options to obtain a splits network with 153 nodes and 165 edges. In the figure inset **(B)**, the input consisted of a subset of 28 taxa and 101 trees (from a splits network with 66 nodes and 72 edges). The recombination events of the sequences in panel B of this figure as determined using RDP4 are shown in Table 4.

Neighbor-net analyses showed likelihood of intra-/inter-lineage recombination events between *S*-gene sequences. Figure 5 represents a networked relationships among 70 *S*-gene sequences (including the 33 sequences from this study). Verification of recombination breakpoints using RDP4 resulted in identification of nine recombination events (Table 4), all of which reflected the results presented in Figure 5. All nine recombination events were statistically (corrected *p*-values of ≤1 × 10 ^−6^) supported by at least six of the seven algorithms used for the analyses. Eight of the recombination events are located in the subunit S2 (in regions containing FP and/or HR1/2 domains), while the recombination event in the field sequence 2598/21 is in the N-terminal receptor domain, which contains HVRs I and II (Table 4).

Three recombination events are in Mass-type, i.e., in the Mass-type/Conn recombinant vaccine 1623/19 sequence, and the field sequences 2860/21 and 2598/21 from Southern and Northern Mexico, respectively. In all three events, both “major parent” sequence (i.e., sequence in other viruses most closely related to the sequence surrounding the recombinant/transferred regions) and “minor parent” sequence (i.e., sequence in other viruses most closely related to the recombinant regions) are Mass-type, with 99.8–100% nt identities between the recombinant region and the “parental” sequences (Table 4). Four other recombination events, all identified in Holte/Iowa-97-like sequences 2731/21 and 2960/21, and Ark-like sequences 2563/21 and 2930/21, all from Central Mexico, have their “parental” sequences in viruses from different lineages (Table 4). One of the recombination events (involving the 4/91 vaccine variant 1619/19 sequence) has field sequence 2833/21 as “major parent” and an unknown “minor” parent inferred to be a 1996 Mass-type vaccine strain (FJ904716/US/Conn46 vaccine/96). In this event, the nt identities between the recombinant region and the “parental” sequences is low (96–96.8%), which probably imply the recombinant regions could have accumulated further mutations, or that the recombination event happened long before the isolation and eventual divergence of the recombinant and parental viruses.

## Discussion

Infectious bronchitis is arguably one of the most important avian respiratory diseases in Mexico, and IBVs are commonly found in flocks, which, despite being vaccinated with the government-approved Mass-type vaccines, exhibit respiratory clinical signs consistent with IB. Ark-like, Mass-type, Holte/Iowa-97, and SAI strains have been previously identified in Mexico using molecular and serological assays (18, 29–31). Most of the Mexican variants have been reported from commercial flocks, but the likelihood of spillover to backyard poultry via introduction of surplus chickens from commercial enterprises, and via use of attenuated vaccines, cannot be ruled out. It is notable that all the IBVs reported in the current study were obtained from clinical samples from apparently healthy flocks. This observation may be due to mild clinical signs. Additionally, a lack of clinical signs of viral infection could be due to persistent (i.e., latent, chronic or slow) infection where the virus is not cleared from infected birds but remains in specific host cells. Although some studies have suggested occurrence of this type of infection for IBVs (70), the phenomenon remains to be convincingly demonstrated.

Studies have demonstrated the versatility of FTA cards as a low-cost option for collection, storage, transportation and preservation of genetic materials from field samples for the surveillance of various viral agents (71–74). Our study demonstrates that, despite the weakness of FTA cards in yielding lower quantities of mostly fragmented RNAs, resulting data is of sufficiently high quality to allow the assembly of full-length viral genomes, even when the clinical samples are prepared under field conditions. This opens up the advantages of FTA cards to NGS-based diagnostics of avian viruses via direct RNA sequencing from field-collected samples without the need of passage in eggs or transportation in liquid media. Avoiding egg-passage is advantageous for several reasons, including biosafety issues (there is no live virus manipulation, amplification, escape issues), and the absence of selective virus amplification (passage may select variants with a minority representation in the host, or introduce genetic changes).

High abundance of host/bacterial RNAs in samples prepared under field conditions, which, from our experiences and those of others, can constitute over 95% of NGS reads (38), results in low sensitivity in detection of viral RNAs. These challenges notwithstanding, our optimized protocols increased NGS sensitivity (high levels of virus-specific reads vs. low levels of host-/bacteria-specific reads), which produced complete genome and/or S-gene sequences (with optimal read coverage depth and coverage). Some of the assembled genome sequences were missing nt at the 5′- and/or 3′- termini, but all coding regions had sufficient depth coverage (≥10X). Furthermore, within single NGS runs (48 multiplexed samples per run), we obtained complete and/partial genome/gene sequences of various RNA viruses belonging to six taxonomic families, some of which were co-isolated with IBV.

Although the general genome organization (5′UTR-[Rep1a-Rep1b-S-3a-3b-E-M-4b-4c-5a-5b-N-6b]-3′UTR) slightly differs from many previously reported IBVs regarding presence of 4b, 4c, and 6b, it is consistent with some IBVs previously reported in various geographical regions across the globe (11, 75–77). The three accessory genes are thought to be strain-dependent/species-specific, but their presence and/or genomic organization are not a prerequisite to production of viable virus progeny, and their role in viral pathogenesis remains undetermined (11, 78). The presence of these auxiliary and apparently non-essential accessory genes is attributable to the IBV's flexible genome, which tolerates not only for stable insertion of novel genes, but also for reorganization (78). The genomic organization of the essential genes is however canonically conserved, including the overlaps between the six genes, except in overlaps between genes 3 and 4 in the 4/91 vaccine variant 1619/19 sequence and the field sequence 2944/21 from Northern Mexico. Most of the variations in the genome organization are in overlaps among the accessory genes and in the presence of VncRNAs in gene 4 (between the structural M and 4b) and gene 6 (between the structural N and the 6b). The sources of gene overlaps remain unknown, but one school of thought is that they result from either utilization of previously unused ORFs to create novel genes, or by adjusting locations of start/stop codons into sequences of existing genes (79).

Both the length and key physical properties at specific positions within the 20-residue region harboring the S1/S2 cleavage motif in all the 33 sequences are evidence that the *S*-glycoprotein of the viruses undergo sufficient furin-mediated cleavage and viral-host membrane fusion (61). In addition to the S1/S2 cleavage, all eight Mass-type, eight Ark-like and the single CAV viruses assembled in this study contain the auxiliary S2'cleavage site downstream the primary S1/S2 cleavage site, which results in a 153-amino acid-long dual-cleavage peptide. Although it does not directly influence IBV pathogenesis, this peptide is postulated to influence the S glycoprotein conformation in some CoVs; deletion of the peptide was demonstrated to affect fusion and recovery of Vero cell-adapted Baudette mutants (8). Although the S2' site is not absolutely required, it is thought to enhance viral infection and replication in some CoVs (80). Nevertheless, it remains unclear why the S2' cleavage site is absent in the sequences belonging to lineages GI-3 (*n* = 2 sequences) and GI-13 (*n* = 14 sequences), regardless of the sample types.

Results of the phylogenetic classification agreed with the comparative pairwise nt sequence homologies between the vaccine vs. field sequences as well as the homologies shared with other IBVs in genomic databases. To the best of our knowledge, 793B- and CAV-like strains have not been previously reported in Mexico. However, there are unpublished reports f the use of 793B vaccines in Mexico. CAVs are indigenously North America currently. CAVs are indigenously North America currently consisting of 12 published viruses from Pennsylvania, California and Alabama, causing respiratory, renal and reproductive diseases (18). Except the 4/91 vaccine variant 1619/19 sequence and the field sequence 2523/21 from Central Mexico, 793B-like sequences are from Southern and Northern Mexico (*n* = 6 sequences each). The only CAV-like sequence is the field sequence 2944/21 from Northern Mexico, which clustered with, among others, isolate MN512437/ck/Can/18-048430/17. The Canadian 18-048430 virus is similar to a DMV strain originally isolated from a virus outbreak in a commercial broiler flock in the Delmarva peninsula in 2011 and the DMV-like strain MK878536/ck/GA9977/19 isolated from broiler chickens in Georgia, USA in 2019 (69, 81). None of the 33 sequences in this study clustered with indigenous SAI viruses based on the *S*-/*S1* and HVRs I-III trees. However, based on complete genome and other specific genomic regions, the SAI viruses clustered in distinct subclades within larger clades containing strains from other lineages, which, coupled with the clustering observed from S-/S1 and HVRs I-III trees, could be interpreted to imply decreased prevalence of the SAI viruses, resulting in the emergence of new, more fit field variants.

Since the coronaviruses can undergo recombination to result in novel variants, and since changes in the spike protein gene can result in shifts in antigenicity and/or tropism, we screened specifically for mutations and recombination in the S-gene (82). We noted various indels and point mutations in the subunit S1 HVRs I-III across the five IBV lineages. The most notable are in the HVR I (residues 38–69), which has been experimentally demonstrated in some IBVs to be critical for binding of S glycoprotein to host's respiratory tract tissues (14). Examples include 2-residue deletions between 60–63 and P63G substitutions in the sequences from Central (2731/21 and 2960/21) and Northern (2944/21) Mexico. Another example is a 3-residue deletion (between positions 69-63) coupled with a P63R substitution in sequence 2598/21 (Northern Mexico). Further, a H43Y (found in the sequence of the 4/91 vaccine variant 1619/19 in our study) was postulated to enhance the fitness of ArkDPI vaccine strain (via efficiency in binding to tracheal membranes) (83). Whether these changes in the S1 spike result in subsequent change in host cell binding, tissue tropism, or evasion of neutralizing antibodies remains to be determined.

The recombination events, presumably between vaccine and field viruses (intra- and inter-lineages), were found in the HVRs 1 and II of subunit S1 (in the field sequence 2598/21) and in the FP, HRs 1 and 2 domains of subunit S2 (in two vaccine and six field sequences). Recombination in the HVRs and in the above-mentioned domains, coupled with the various indels and mutations found in these regions, could modulate the S glycoprotein fusion properties, with the potential of variants to not only broaden their tissue-tropism (and possibly host-range), but also to efficiently adapt to naïve hosts as previously suggested (82). Additionally, these variants can potentially be a result of immune selection pressures (36). An example of this is the DE072 vaccine, which when first identified, showed more relatedness to a Dutch variant that to the US variants, but after undergoing a decade-long use, the strain evolved to a poor vaccine candidate (24).

## Conclusions

This study has demonstrated FTA cards as adoptable, low-cost option for untargeted discovery and full-length sequencing of avian viruses from field-collected clinical samples from various tissues. Our data demonstrates that multiple distinct IBV serotypes/strains are co-circulating in Mexican commercial chickens, with the high likelihood of intra- and inter-lineage recombination, as well as indels and point mutations which are potentially driving the generation of new subpopulations of field variants capable spreading and adapting to chicken populations in the country. It should be further investigated whether strains emerging from the commercial enterprises may have spilled over to backyard poultry, and whether they have further evolved into strains that are distantly related to the predominantly SAI strains. More importantly, our data reiterates the need for enhanced surveillance of IBVs in Mexico and the Latin America region, as well as a review of the vaccines currently used in the control of IBVs.

## Data Availability Statement

The datasets presented in this study can be found in online repositories. The names of the repository/repositories and accession number(s) can be found below: https://www.ncbi.nlm.nih.gov/genbank/, OM912676, OM912677, OM912678, OM912679, OM912680, OM912681, OM912682, OM912683, OM912684, OM912685, OM912686, OM912687, OM912688, OM912689, OM912690, OM912691, OM912692, OM912693, OM912694, OM912695, OM912696, OM912697, OM912698, OM912699, OM912700, OM912701, OM912702, OM912703, OM912704, OM912705, OM912706, OM912707, and OM912708.

## Author Contributions

CA, ED, and HK: conceptualization. CA, DS, and ED: funding acquisition. DS, SL, NC, and ED: project administration and coordination. HK: methodology and writing—original draft. HK, JV, and CL: data curation and formal analyses. CL, DS, and CA: writing—review and editing. All authors read and agreed to the published version of the manuscript.

## Funding

This research was funded by Agricultural Research Service (ARS), USDA CRIS, and Oak Ridge Institute for Science and Education (ORISE) postdoctoral appointment. The funders had no role in data analyses or interpretation, or in the writing of the manuscript.

## Conflict of Interest

JV and CA were employed by BASE_2_BIO. The remaining authors declare that the research was conducted in the absence of any commercial or financial relationships that could be construed as a potential conflict of interest.

## Publisher's Note

All claims expressed in this article are solely those of the authors and do not necessarily represent those of their affiliated organizations, or those of the publisher, the editors and the reviewers. Any product that may be evaluated in this article, or claim that may be made by its manufacturer, is not guaranteed or endorsed by the publisher.

## Supplementary Material

Supplementary Table 1 Background information and sequencing data of the 55 samples in which IBV RNA was detected in this study. The quantification of the dsDNA (HiFi PCR products), sequencing libraries, proportions of the host (chicken) reads, and the IBV-specific reads obtained from the NGS data are shown. Complete genome sequences (*n* = 33), partial genome sequences (*n* = 6), complete *S*-gene sequences (*n* = 5), and partial *S*-gene sequences (*n* = 11) were assembled from NGS reads in the current study. Other identified microbial agents (viral and bacterial) that are of avian interest based on literature and expert knowledge are listed in the table.

Supplementary Table 2 Genes, coding regions and nt lengths of the 33 IBV sequences identified in this study. The nt lengths (and gene intervals in brackets) of each of the genes and the 5'-/3'-UTRs are shown.

Supplementary Table 3 The 15 accessory proteins (2–15) that are proteolytically processed from Rep1ab by the virally-encoded PL ^pro^ and 3CL ^pro^. For each of the 33 IBV sequences assembled in this study, the size (amino acid) of each cleavage product are indicated. The position (amino acid residue) of each product on the Rep1a (2–11) and Rep1b (12–16) are shown in brackets.

Supplementary Table 4 Analysis of the *S*-gene nt sequences assembled in this study. The arrow (↓) indicates the S1/S2 proteolytic cleavage site. The conserved cysteine-flanked region in the S2 is indicated (amino acid residues in bold and underlined). The numbers in subscript indicate the position of the amino acid residues in the S protein sequence.

Supplementary Table 5 Comparative pairwise homologies of IBV gene nt sequences between the field and vaccine sequences assembled in this study. Field sequences with the highest pairwise homologies to the vaccine sequences are in bold font, while sequences with the lowest are underlined. The lineage (serotype) classification is based on *S1*-gene sequence according to Valastro et al. (18).

Supplementary Table 6 BLASTn results of the 33 complete genome nt sequences assembled in this study. The lineage (serotype) classification is based on S1-gene sequence according to Valastro et al. (18). GenBank accession numbers are shown for the best BLASTn hit for each sequence.

Supplementary Figure 1 Maximum likelihood phylogenetic tree of nt sequences of the envelope (E), membrane (M) and nucleocapsid (N) genes using T92 model in MEGA 6. The 3 vaccine sequences assembled in this study are highlighted in blue color; the 30 field sequences are color-coded based on sampling regions in Mexico. The analysis involved 74 sequences. All positions with <95% site coverage were eliminated. The final datasets for the *E, M*, and *N* genes had 304, 659, and 1,215 positions, respectively.

Supplementary Figure 2 Maximum likelihood phylogenetic tree of nt sequences of accessory genes in (A) gene 3 (3a/3b), gene 5 (5a/5b) and gene 6 (6b) using T92 model in MEGA 6. The 3 vaccine sequences assembled in this study are highlighted in blue color; the 30 field sequences are color-coded based on sampling regions in Mexico. The analysis involved 74 (3a/b and 5a/b) and 63 (6b) sequences. All positions with less than 95% site coverage were eliminated. The final datasets for the 3a/b, 5a/b, and 6 genes had 365, 443, and 130 positions, respectively.
