# Supplementary material for: Genome Sequence Variations of Infectious Bronchitis Virus Serotypes From Commercial Chickens in Mexico
Source: Front Vet Sci. 2022 Jul 12;9:931272. doi: 10.3389/fvets.2022.931272 (PMC9315362; doi:10.3389/fvets.2022.931272)
Supplement: Supplementary Table 6 — BLASTn results of the 33 complete genome nt sequences assembled in this study. The lineage (serotype) classification is based on S1-gene sequence according to Valastro et al. (18). GenBank accession numbers are shown for the best BLASTn hit for each sequence. [file Table_6.docx]

Supplementary Material

Genome Sequence Variations of Infectious Bronchitis Virus Serotypes from Commercial Chickens in México

Henry M. Kariithi ^1,2^, Jeremy D. Volkening^3^, Christina M. Leyson ^1^, Claudio L. Afonso ^3^, Nancy Christy ^4^, Eduardo L. Decanini ^4^, Stéphane Lemiere ^5^, David L. Suarez ^1*^

^1^ Exotic and Emerging Avian Viral Diseases Research Unit, Southeast Poultry Research Laboratory, U.S. National Poultry Research Center, USDA-ARS, Athens, GA, USA.

^2^ Biotechnology Research Institute, Kenya Agricultural and Livestock Research Organization, Kaptagat Rd, Nairobi, Kenya.

^3^BASE_2_BIO, Oshkosh, WI, USA

^4^ Boehringer Ingelheim Animal Health, México; ^5^ Boehringer Ingelheim Animal Health, France.

*** Correspondence:** David L. Suarez ([david.suarez@usda.gov](mailto:david.suarez@usda.gov))

**Supplementary file 6:** **Table S6: BLASTn results of the 33 complete genome nucleotide sequences assembled in this study**. The lineage (serotype) classification is based on S1-gene sequence (Valastro *et al*., 2016). GenBank accession numbers are shown for the best BLASTn hit for each sequences.

| **sequence** | **sampling date** | **origin (region)** | **flock age (days/weeks)** | **tissue** | **best BLASTn hit (isolate)** | **lineage (serotype)** | **seq. length (*excluding poly(A) tail*)** | **Hit start** | **Hit end** | **query coverage** | **Bit-Score** | **identity** |
| --- | --- | --- | --- | --- | --- | --- | --- | --- | --- | --- | --- | --- |
| OM912698/live mass-type vaccine strain/1616/19 | 23-Apr-19 | n/a | vaccine | n/a | MK937828/ck/CN/I1124/16 | GI-1 (Mass-type) | 27,469 | 1 | 27,459 | 99.96% | 50,314.9 | 99.74% |
| OM912697/4/91 vaccine variant strain/1619/19 | 23-Apr-19 | n/a | vaccine | n/a | MN548285/ck/UK/CR88/11 | GI-13 (793B) | 27,656 | 2 | 27,647 | 99.96% | 51,025.9 | 99.98% |
| OM912696/Mass-type-Conn recombinant vaccine strain/1623/19 | 23-Apr-19 | n/a | vaccine | n/a | MK937828/ck/CN/I1124/16 | GI-1 (Mass-type) | 27,474 | 3 | 27,459 | 99.95% | 47,753.6 | 98.06% |
| OM912680/ck/MEX/2353/20 | 25-Nov-20 | South | broiler (42D) | choanal/lung | MZ367369/ck/BE/4134 001/19 | GI-13 (793B) | 27,140 | 193 | 27,461 | 99% | 49961 | 99.93% |
| OM912682/ck/MEX/2354/20 | 25-Nov-20 | South | broiler (42D) | spleen/bursa | MZ367369/ck/BE/4134 001/19 | GI-13 (793B or 4/91) | 27,624 | 1 | 27,598 | 9.91% | 50915.9 | 99.97% |
| OM912678/ck/MEX/2359/20 | 25-Nov-20 | South | broiler (42D) | choanal/lung | MH779860/ck/USA/Ark99 pathogenic field str./14 | GI-9 (Ark-like) | 27,634 | 1 | 27,603 | 99% | 42126 | 94.7% |
| OM912677/ck/MEX/2360/20 | 25-Nov-20 | South | broiler (42D) | spleen/bursa | MH779860/ck/USA/Ark99 pathogenic field str./14 | GI-9 (Ark-like) | 27,167 | 1 | 27,510 | 99% | 41184 | 94.23% |
| OM912695/ck/MEX/2523/21 | 28-Jan-21 | Central | broiler (29D) | choanal/lung | MT665806/ck/Canada/17-038913/17 | GI-13 (793B or 4/91) | 27,449 | 32 | 27,471 | 99.96% | 39,962.60 | 92.96% |
| OM912694/ck/MEX/2562/21 | 25-Feb-21 | Central | broiler (28D) | choanal/lung | MH779860/ck/USA/Ark99 pathogenic field str./14 | GI-9 (Ark-like) | 27,671 | 12 | 27,603 | 99.68% | 42,014.2 | 94.16% |
| OM912693/ck/MEX/2563/21 | 25-Feb-21 | Central | broiler (28D) | spleen/bursa | MH779860/ck/USA/Ark99 pathogenic field str./14 | GI-9 (Ark-like) | 27,805 | 1 | 27,125 | 97.52% | 41,567 | 94.34% |
| OM912692/ck/MEX/2592/21 | 1-Apr-21 | South | broiler (21D) | choanal/lung | MZ367369/ck/BE/4134_001/19 | GI-13 (793B or 4/91) | 27,700 | 1 | 27,601 | 99.64% | 50,911.4 | 99.96% |
| OM912685/ck/MEX/2598/21 | 1-Apr-21 | North | broiler (27D) | spleen/bursa | MK937832/ck/CH/LJL/130906/13 | GI-1 (Mass-type) | 27,095 | 26 | 27,334 | 99% | 42,984 | 96.98% |
| OM912684/ck/MEX/2602/21 | 1-Apr-21 | South | broiler (21D) | spleen/bursa | KY626045/BR/Ma5/16 | GI-1 (Mass-type) | 27,623 | 1 | 27,619 | 99% | 50,837.5 | 99.89% |
| OM912691/ck/MEX/2721/21 | 6-Jun-21 | North | broiler (21D) | choanal/lung | KP118881/ck/CH/LBJ/140413/14 | GI-13 (793B or 4/91) | 27,280 | 56 | 27,458 | 99% | 48,107 | 99.42% |
| OM912690/ck/MEX/2723/21 | 6-Jun-21 | North | broiler (28D) | choanal/lung | KP118881/ck/CH/LBJ/140413/14 | GI-13 (793B or 4/91) | 27,694 | 178 | 27,596 | 98% | 48,898 | 99.52% |
| OM912689/ck/MEX/2725/21 | 6-Jun-21 | North | broiler (21D) | choanal/lung | MZ367369/ck/BE/4134_001/19 | GI-13 (793B or 4/91) | 27,594 | 1 | 27,193 | 99.91% | 43,908 | 95.33% |
| OM912676/ck/MEX/2731/21 | 23-Jun-21 | Central | broiler (23D) | choanal/lung | MH779860/ck/USA/Ark99 pathogenic field str./14 | GI-9 (Ark-like) | 27,189 | 193 | 27,603 | 99% | 39,796 | 92.99% |
| OM912688/ck/MEX/2742/21 | 11-Jul-21 | South | broiler (28D) | spleen/bursa | MH779860/ck/USA/Ark99 pathogenic field str./14 | GI-9 (Ark-like) | 27,625 | 10 | 27,625 | 98.83% | 41,873 | 94.07% |
| OM912683/ck/MEX/2743/21 | 11-Jul-21 | South | broiler (28D) | choanal/lung | KY626045/BR/Ma5/16 | GI-1 (Mass-type) | 27,509 | 138 | 27,629 | 100% | 50,446 | 99.69% |
| OM912687/ck/MEX/2748/21 | 15-Jul-21 | South | broiler (28D) | choanal/lung | KY626045/BR/Ma5/16 | GI-1 (Mass-type) | 27,619 | 1 | 27,619 | 100% | 50,843.1 | 99.89% |
| OM912679/ck/MEX/2752/21 | 20-Jul-21 | South | broiler (21D) | choanal/lung | MZ367369/ck/BE/4134_001/19 | GI-13 (793B or 4/91) | 27,022 | 185 | 27,530 | 99% | 49,532 | 99.87% |
| OM912686/ck/MEX/2753/21 | 20-Jul-21 | South | broiler (28D) | spleen/bursa | MZ367369/ck/BE/4134_001/19 | GI-13 (793B or 4/91) | 27,450 | 167 | 27,601 | 99.95% | 50,627 | 99.97% |
| OM912681/ck/MEX/2754/21 | 20-Jul-21 | South | broiler (21D) | choanal/lung | KY626045/BR/Ma5/16 | GI-1 (Mass-type) | 27,410 | 1 | 25,041 | 99% | 50,172 | 99.63% |
| OM912699/ck/MEX/2818/21 | 9/10/2021 | North | broiler (28D) | choanal/lung | KP118881/ck/CH/LBJ/140413/14 | GI-13 (793B or 4/91) | 27,616 | 1 | 27,099 | 98.07% | 48,327.9 | 98.87% |
| OM912700/ck/MEX/2819/21 | 9/10/2021 | North | broiler (28D) | spleen/bursa | KP118881/ck/CH/LBJ/140413/14 | GI-13 (793B or 4/91) | 27,638 | 1 | 27,099 | 98% | 48,311.3 | 98.86% |
| OM912701/ck/MEX/2826/21 | 9/13/2021 | South | broiler (28D) | choanal/lung | MZ367369/ck/BE/4134_001/19 | GI-13 (793B or 4/91) | 27,567 | 31 | 27,595 | 99.99% | 50,772.9 | 99.91% |
| OM912702/ck/MEX/2833/21 | 9/27/2021 | North | broiler (21D) | choanal/lung | MZ367369/ck/BE/4134_001/19 | GI-13 (793B or 4/91) | 27,694 | 1 | 27,587 | 99% | 43,943 | 95.4% |
| OM912703/ck/MEX/2860/21 | 10/12/2021 | South | broiler (28D) | choanal/lung | KY626045/BR/Ma5/16 | GI-1 (Mass-type) | 27,600 | 31 | 27,630 | 99% | 50,190 | 99.48% |
| OM912704/ck/MEX/2930/21 | 23-Nov-21 | Central | broiler (21D) | choanal/lung | MH779860/ck/USA/Ark99 pathogenic field str./14 | GI-9 (Ark-like) | 27,749 | 1 | 27,603 | 99.47% | 43,052.1 | 99.82% |
| OM912705/ck/MEX/2944/21 | 7-Dec-21 | North | broiler (28D) | choanal/lung | MN512437/ck/Can/18-048430/17 | GI-17 (CAV) | 27,558 | 136 | 27,683 | 99.92% | 44,819 | 96.04% |
| OM912706/ck/MEX/2956/21 | 14-Dec-21 | South | broiler (28D) | choanal/lung | MH779860/ck/USA/Ark99 pathogenic field str./14 | GI-9 (Ark-like) | 27,682 | 1 | 27,603 | 99% | 41,929 | 94.09% |
| OM912707/ck/MEX/2960/21 | 14-Dec-21 | Central | layer (7.6 W) | spleen/bursa | MH779860/ck/USA/Ark99 pathogenic field str./14 | GI-9 (Ark-like) | 27,526 | 1 | 27,564 | 98% | 40,734 | 93.39% |
| OM912708/ck/MEX/2961/21 | 15-Dec-21 | Central | broiler (21D) | choanal/lung | MH779857/ArkGAP20 vaccine/15 | GI-9 (Ark-like) | 27,577 | 8 | 27,602 | 99.98% | 41,297.7 | 93.7% |
