# Supplementary figures and images for: Genome Sequence Variations of Infectious Bronchitis Virus Serotypes From Commercial Chickens in Mexico
Source: Front Vet Sci. 2022 Jul 12;9:931272. doi: 10.3389/fvets.2022.931272 (PMC9315362; doi:10.3389/fvets.2022.931272)

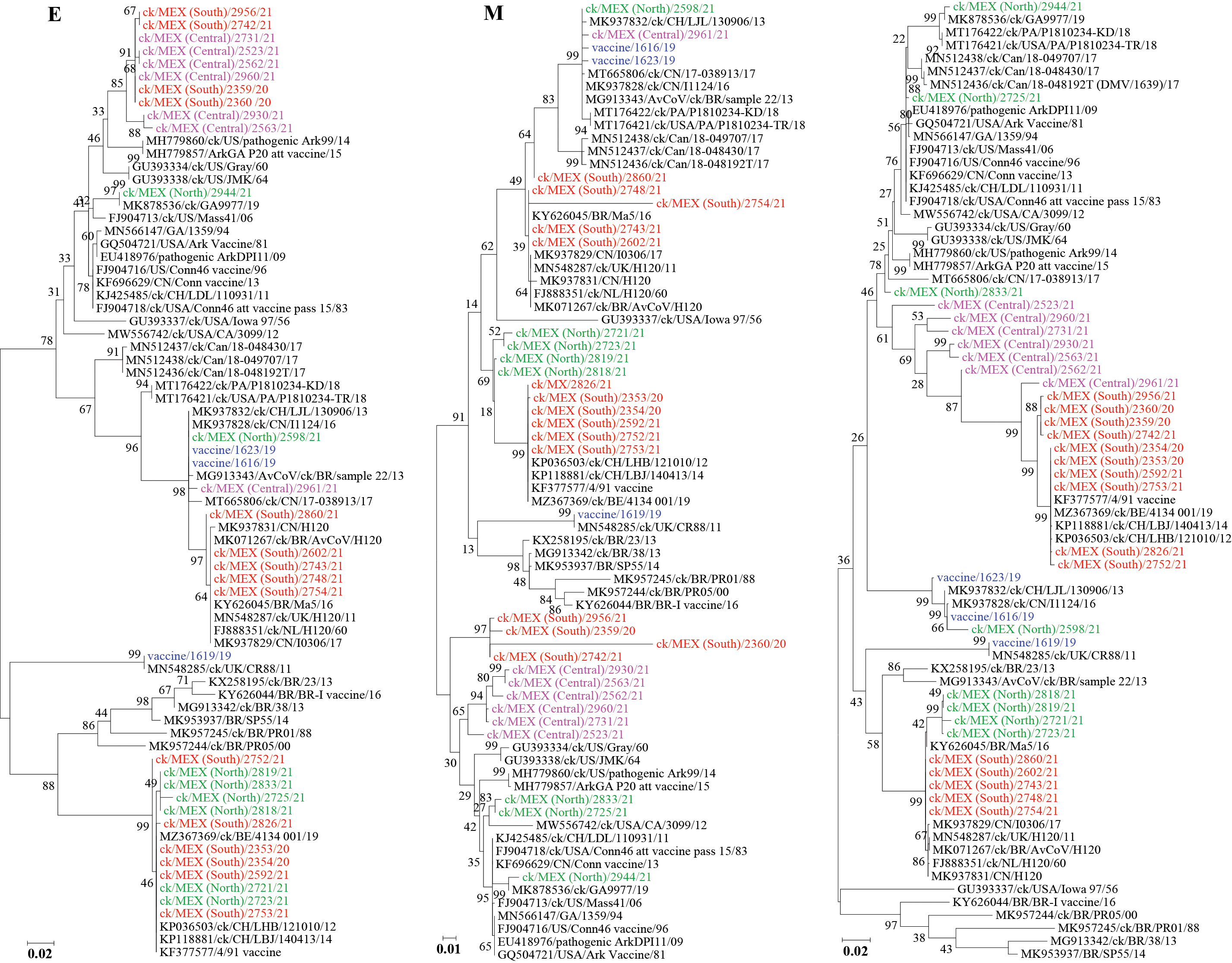

Supplement: Supplementary Figure 1 — Maximum likelihood phylogenetic tree of nt sequences of the envelope (E), membrane (M) and nucleocapsid (N) genes using T92 model in MEGA 6. The 3 vaccine sequences assembled in this study are highlighted in blue color; the 30 field sequences are color-coded based on sampling regions in Mexico. The analysis involved 74 sequences. All positions with <95% site coverage were eliminated. The final datasets for the E, M, and N genes had 304, 659, and 1,215 positions, respectively. [file Image_1.JPEG]

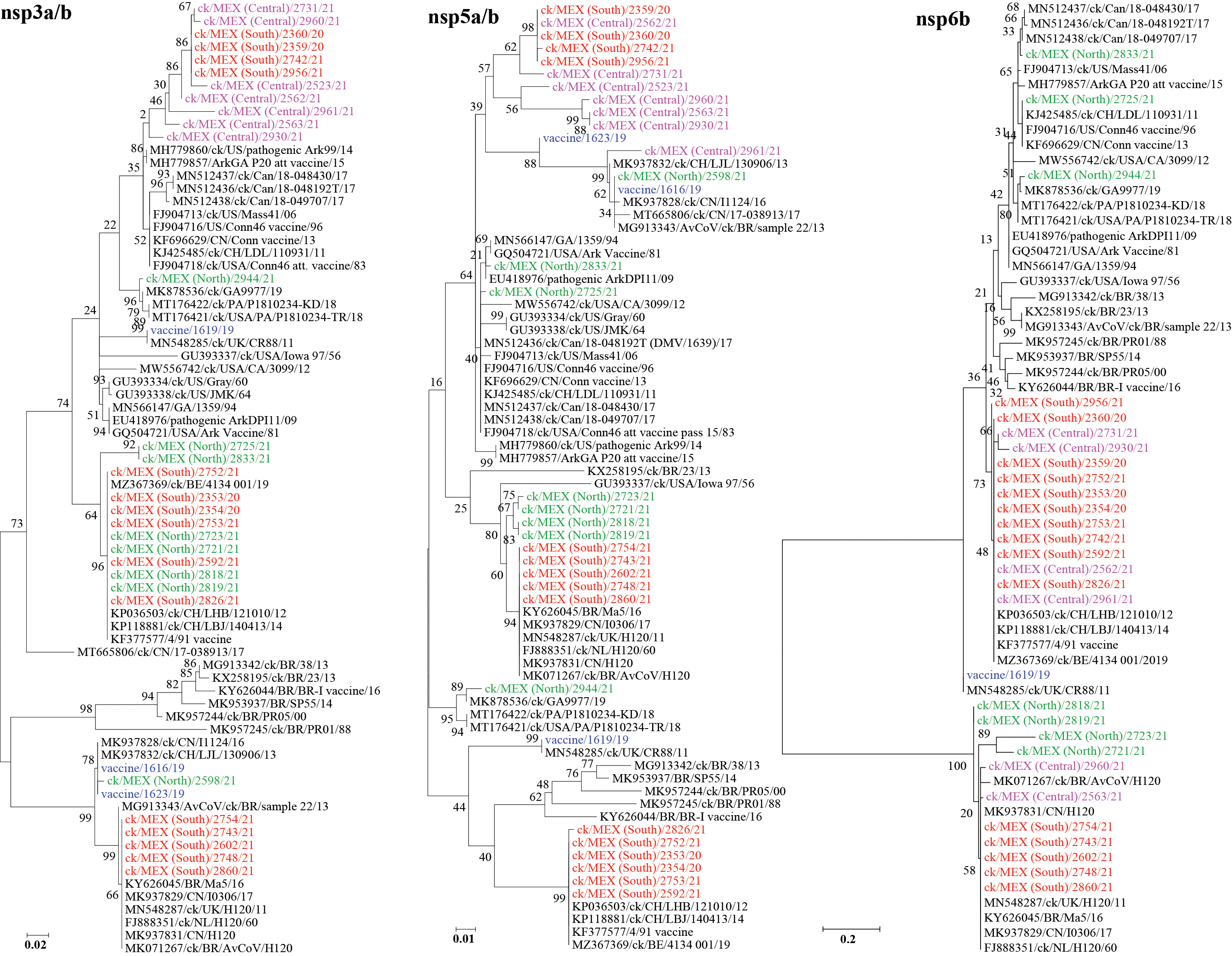

Supplement: Supplementary Figure 2 — Maximum likelihood phylogenetic tree of nt sequences of accessory genes in (A) gene 3 (3a/3b), gene 5 (5a/5b) and gene 6 (6b) using T92 model in MEGA 6. The 3 vaccine sequences assembled in this study are highlighted in blue color; the 30 field sequences are color-coded based on sampling regions in Mexico. The analysis involved 74 (3a/b and 5a/b) and 63 (6b) sequences. All positions with less than 95% site coverage were eliminated. The final datasets for the 3a/b, 5a/b, and 6 genes had 365, 443, and 130 positions, respectively. [file Image_2.JPEG]
